# *ABI1*‐based expression signature predicts breast cancer metastasis and survival

**DOI:** 10.1002/1878-0261.13175

**Published:** 2022-01-26

**Authors:** Angelina Regua, Csaba Papp, Andre Grageda, Baylee A. Porter, Tiffany Caza, Isabelle Bichindaritz, Mira Krendel, Abirami Sivapiragasam, Gennady Bratslavsky, Vladimir A. Kuznetsov, Leszek Kotula

**Affiliations:** ^1^ Department of Urology SUNY Upstate Medical University Syracuse NY USA; ^2^ Department of Biochemistry and Molecular Biology SUNY Upstate Medical University Syracuse NY USA; ^3^ Department of Pathology SUNY Upstate Medical University Syracuse NY USA; ^4^ Department of Computer Science SUNY Oswego NY USA; ^5^ Department of Cell and Developmental Biology SUNY Upstate Medical University Syracuse NY USA; ^6^ Department of Medicine SUNY Upstate Medical University Syracuse NY USA; ^7^ Present address: Department of Cancer Biology Wake Forest University School of Medicine Winston‐Salem NC 27101 USA

**Keywords:** *Abi1*, breast cancer, metastasis, preclinical mouse, survival signature, WAVE

## Abstract

Despite the current standard of care, breast cancer remains one of the leading causes of mortality in women worldwide, thus emphasizing the need for better predictive and therapeutic targets. ABI1 is associated with poor survival and an aggressive breast cancer phenotype, although its role in tumorigenesis, metastasis, and the disease outcome remains to be elucidated. Here, we define the *ABI1*‐based seven‐gene prognostic signature that predicts survival of metastatic breast cancer patients; *ABI1* is an essential component of the signature. Genetic disruption of *Abi1* in primary breast cancer tumors of PyMT mice led to significant reduction of the number and size of lung metastases in a gene dose‐dependent manner. The disruption of *Abi1* resulted in deregulation of the WAVE complex at the mRNA and protein levels in mouse tumors. In conclusion, ABI1 is a prognostic metastatic biomarker in breast cancer. We demonstrate, for the first time, that lung metastasis is associated with an *Abi1* gene dose and specific gene expression aberrations in primary breast cancer tumors. These results indicate that targeting ABI1 may provide a therapeutic advantage in breast cancer patients.

## Graphical abstract

Breast cancer (BC) remains one of the leading causes of mortality in women worldwide. We identified a set of seven genes that predicted the metastatic potential of primary BC tumors. Additionally, we confirmed the role of *ABI1* that we identified as a key prognostic metastatic biomarker, in a mouse model of BC metastasis to the lungs. Our data showed that the *ABI1*‐seven‐gene signature holds prognostic potential and that targeting of *ABI1* may provide therapeutic advantage in patients with BC.

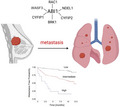

**Abbreviations**
ABI1/2/3: abelson interactor protein‐1, −2, −3
ABL: tyrosine‐protein kinase ABL1
ARP2/3: actin‐related proteins ARP2 and ARP3
BRK1: BRICK1 subunit of SCAR/WAVE actin nucleating complex
CK14: cytokeratin 14
CK8: cytokeratin 8
CNA: copy number alteration
CYFIP: cytoplasmic FMR1‐interacting protein
DDg: data‐driven grouping
DFS: disease‐free survival
DMFS: distant metastasis‐free survival
ECL: enhanced chemiluminescence
HSPC300: hematopoietic stem/progenitor cell protein 300
KO: knockout
MAPK: mitogen‐activated protein kinase
MEF: mouse embryonic fibroblast
MMTV: mouse/murine mammary tumor virus
NCKAP1: NCK‐associated protein 1
OS: overall survival
PCA: principal component analysis
PI3K/AKT: phosphoinositide 3‐kinase/protein kinase B
PMF: primary myelofibrosis
PyMT: polyoma middle T antigen
RT: room temperature (25 °C)
SRA: steroid receptor RNA activator
SWVg: statistically weighted voting grouping
TEB: terminal end buds
WAVE: Wiskott‐Aldrich Verprolin homologous protein

## 1 Introduction

Breast cancer is the most commonly diagnosed noncutaneous cancer in American women, causing an estimated 200 000 deaths and over 40 000 new diagnoses each year [1]. Despite current treatment modalities that combine surgical intervention, radiation, and adjuvant chemotherapy, many patients relapse after years of treatment and present with metastatic and often incurable diseases. Metastasis of breast tumors accounts for the majority of breast cancer‐related deaths [2]. Thus, there is an urgent need to identify novel molecular targets for the development of new treatments against breast cancer.

The critical role of actin polymerization in breast tumor progression and invasion is well established, but the underlying mechanisms remain to be elucidated. Candidate mechanisms of tumor progression involving actin include cell–matrix interactions, invadopodia formation, and increased cell motility, which can all be attributed to increased actin polymerization in invading cells [3, 4]. The WAVE complex is a heteropentameric nucleation‐promoting factor of F‐actin polymerization and comprises WAVE proteins (1/2/3), Abelson interactor (1/2/3), SRA1/CYFIP1, NAP1, and BRK1/HSPC300 [5, 6, 7]. These proteins are encoded by genes *WASF(1,2,3)*, *ABI* (1/2/3), *CYFIP(1*,*2)*, *NCKAP1*, *and BRK1*, respectively [7]. The WAVE regulatory complex in response to RAC1 activation has been proposed to act as a regulator of cell motility by promoting ARP2/3‐dependent actin polymerization at the leading cell edge [8, 9]. Importantly, increased levels of ARP2/3 and WAVE2 are correlated with an increased risk of invasive breast cancer [10].

The integrity and activity of the WAVE complex are reliant on the presence of all complex members; the loss of any single constituent can lead to altered cell phenotypes [11]. Upstream pathway signaling partners of WAVE complex such as RAC1 [6, 12, 13] and NUDEL modify its activity [14]. Abelson interactor 1 (ABI1) is crucial for WAVE complex stability and regulation of specific actin‐dependent processes such as cell motility and adhesion, macropinocytosis, and embryonic development [15, 16, 17]. Our previous studies demonstrated that constitutive *Abi1* loss results in murine embryonic lethality [16]. ABI1 is an adaptor protein that promotes phosphorylation of substrates, such as WAVE2, by ABL kinase and has also been shown to be important for capping of F‐actin filaments, thus highlighting its regulatory role in cellular homeostasis and actin turnover [18]. WAVE1 and WAVE2 have differential roles in actin polymerization output resulting in distinct effect on actin meshwork at the plasma membrane [19, 20].

In cancers, WAVE complex’s molecular composition is dynamic and can be represented by distinct molecular subcomplexes due to deregulation of component levels [7, 11, 14]. Furthermore, several cell context‐dependent WAVE/ABI1 subcomplexes can form and exhibit distinct functions activated and maintained through different mechanisms [7, 11, 14, 20]. For instance, enhanced levels of *WASF3* gene expression could promote cancer cell invasiveness and are associated with the highly aggressive breast cancer subtypes [21, 22]. However, recent studies also demonstrate potential tumor suppressor function of *Wasf3* upon overexpression in PyMT breast cancer cells [22] thus indicating heterogeneity of WAVE3‐based complex signaling through differential effect on actin cytoskeleton and cell proliferation [23, 24].

WAVE complex dysregulation in cancer provides input into cell cycle progression and warrants the study of its role in breast cancer [25]. Although the specific molecular mechanism has yet to be uncovered, WAVE2 was linked to regulation of cell cycle progression through RAC1, Arp2/3, and ARPIN. Upregulation of the Arp2/3 subunit, ARPC1B, is associated with very poor metastasis‐free survival of breast cancer patients, but inhibition of ARP2/3 prevents cycle progression through RAC1 transformation [7, 25].

Alterations in *ABI1* expression have been associated with tumor initiation and progression in human cancers, thus indicating that ABI1 protein levels must be tightly regulated in cells. *ABI1* dysregulation has been implicated in several cancers, such as breast, brain, colon, stomach, ovarian, and prostate cancers [26, 27, 28, 29]. Notably, the role of *ABI1* in cancer is not always the same; in some cancers, such as PMF, glioblastoma, and prostate cancer, *ABI1* expression is downregulated [26, 27, 30, 31], whereas in breast cancer, *ABI1* expression is enhanced [32], thus suggesting the tissue and disease‐involving pathway specificity of the role of *ABI1* in oncogenic transformation and indicating the importance of mechanistic studies. The important role of ABI1 in breast cancer has been established in clinical samples. Previously, immunohistochemical studies of over 900 human breast tumor samples showed that *ABI1* overexpression is positively correlated with poor survival and a shorter relapse time in human breast cancer patients [32]. Indeed, the analysis revealed that invasive breast tumors have higher ABI1 protein expression than poorly invasive tumor samples and that increased ABI1 protein levels are significantly correlated with earlier recurrence and shortened survival. These findings have been supported by xenograft models of highly aggressive breast cancer cells (MDA‐MB‐231) lacking *ABI1*, which were unable to grow into large tumors in immunocompromised mice [33]. Taken together, previous data suggest that *ABI1* plays a driving role in the progression of metastatic breast cancers 32, 33, 34.

Several *in vitro* studies have shown the impact of ABI1 in driving breast cancer cell motility, division, and invasiveness; however, its exact role during *in vivo* tumor initiation, progression, and metastasis remains to be elucidated. Thus, we aimed to study the impact of *Abi1* loss on mammary tumor initiation and progression using the polyoma middle T (PyMT) breast cancer mouse model. The PyMT antigen is a transmembrane scaffolding protein with key tyrosine residues that, upon phosphorylation, can activate signaling pathways involved in cell proliferation and survival (e.g., PI3K/AKT and MAPK), making it a reliable model for aggressive breast tumor formation [35]. The PyMT breast cancer model has been well characterized and recapitulates human breast cancer pathology, especially that of the triple‐negative subtype [36].

High levels of ABI1 have been associated with the risks of metastasis of primary tumors and breast cancer mortality, as well as associated with the metastatic phenotype of human breast cancer cell lines *in vitro* [3, 32, 34, 37, 38]. The deficiency of ABI1 has been shown to reduce cell migration and invasiveness of aggressive breast cancer cells and is associated with activity in pathways such as PI3 kinase/AKT and SRC [32, 33].

Here, we define the ABI1‐associated gene expression signature, which predicts the disease metastasis‐free survival (DMFS) of patients with primary breast cancer. The signature includes a subset of WAVE complex genes (*ABI1*, *BRK1*, *WASF3*, *CYFIP1*, *CYFIP2)*, and the direct interactors of WAVE complex (*RAC1* and *NDEL1*). *ABI1* is an essential component of the signature. To model the role *of Abi1* in breast cancer tumor progression and metastasis, we conditionally depleted *Abi1* gene expression in the mammary epithelium of PyMT breast cancer mice using the mammary‐specific Cre recombinase mouse. Our analysis shows that *Abi1* knockout (KO) mice, both with homozygous and heterozygous deletion had more diverse tumor growth kinetics compared to the controls. In KO animals, a significant proportion (between 54% and 64%) of the primary tumors grew slower or not at all. However, the number of identified metastatic foci in lung and their size were significantly reduced in both homozygous and heterozygous KO mice, with the more significant metastasis suppression effect observed in the former. These results indicate that *Abi1* gene dosage in primary tumors is critical for the progression of metastasis in breast cancer. Western blotting analysis of primary tumors supports our previous findings that ABI2 protein expression is increased in animals with homozygous deletion of *Abi1*. Collectively, both our analyses utilizing both human breast cancer gene expression data and genetically engineered *Abi1* knockout breast cancer mouse models support the critical role of ABI1 and *ABI1*‐based gene prognostic signature as novel biomarkers of breast cancer metastases.

## 2 Methods

### 2.1 Reannotation and legacy comparison microarray datasets

The updated and reannotated Rosetta microarray dataset [39] and the Metadata dataset [40, 41] were used for the statistical testing and survival prediction analyses. The Metadata dataset is comprised of Uppsala and Stockholm data cohorts, which totals 249 samples (Affymetrix U133A, U133B) [40, 41]. Rosetta expression microarray dataset of 295 primary breast cancer samples has been downloaded [39] and reprocessed. Probe sequences (60 bp) obtained from the Rosetta dataset were aligned using NCBI’s command line *blastn* program with the following arguments: *‐reward 2 ‐penalty −3 ‐word_size 11 ‐gapopen 5 ‐gapextend 2*. Coordinates with the most significant e‐value were used for each sequence. Ensembl GRCh38.p13 was used to annotate each probe’s given genome coordinates. RefSeq gene symbols were used to annotate probes that were not annotated by Ensembl and contained RefSeq IDs. A total of 32439 expression data points are present with 24479 unique probes (GSE159956).

Our newly updated Rosetta probe set annotation was compared to the original probe set annotation. Our newly updated Rosetta probe set annotation was compared to the original probe set annotation. A total of 11847/24479 (48.4%) of our probe’s gene symbols exactly matched the original gene symbol. A total of 804/24479 (3.2%) probes that have identical gene symbols are on the opposite strand of the given gene. Of the 12632/24479 (51.6%) unique probes that were not an exact match, there were instances where the original set either had a false negative, a false positive, or an alternative gene symbol was used (Fig. S1). For an example of a false negative, see probe ‘Contig44690_RC’. In the original probe set annotation, there is no gene symbol for this probe. However, we found that the gene symbol for this probe was *PTEN*, which was confirmed with the UCSC genome browser.

A total of 7375/24479 (30.1%) probes matched this characteristic. For an example of a false positive, see probe ‘Contig52193_RC’. In the original probe set annotation, there is a gene symbol present for this probe. However, we found that the location of the probe is neighboring the gene body rather than overlapping the gene body. A total of 92/24479 (0.003%) probes matched this characteristic. The remaining percentage of probes that do not exactly match are either instances where the official gene symbol has been updated or instances where a single probe maps to a locus containing multiple genes. For an example of an updated gene symbol, see probe ‘NM_017546’. The original states this probe maps to gene *C40*; however, our method maps this probe to *CNOT11*. Using GeneCards, we found that *CNOT11* and *C40* are the same genes. For an example of a probe that maps to multiple genes, see probe ‘NM_006340’. This probe maps to both *BAIAP2* and *AATK*. The original probe annotation only links this probe to *BAIAP2*. Overall, we improved upon the original probe set annotation by providing gene symbols for a significant portion of false negatives (Fig. S1). Additionally, we show increased consistency of ABI1 expression values between groups following KS‐weighted means batch effect correction (Fig. S2).

### 2.2 Characterization of ABI1 expression, copy number alterations, and associations with breast cancer clinical data

To analyze ABI1 expression, copy number alterations and associations of these characteristics with breast cancer clinical data, we used The Molecular Taxonomy of Breast Cancer International Consortium(METABRIC) ([42] observed in cBioPortal for Cancer Genomics. https://www.cbioportal.org/study/summary?id=brca_metabric). ABI1 profiles from 1904 breast cancer patients including microarray expression, copy number variation, and clinical and cancer samples were downloaded and analyzed.

### 2.3 Survival prediction analysis and multigene prognostic signature identification

We used our data‐driven grouping (DDg) methods (one‐dimensional (univariate), 1D‐DDg, two‐dimensional (bivariate) 2D‐DDg), and statistically weighted voting grouping (SVWg) algorithms for patient's risk stratification onto two and three survival groups representing Kaplan–Meier survival functions (K‐M functions) [37, 43, 44, 45]. These are well‐established statistically based computational methods to identify optimized cutoff values of high‐dimensional variable domains that transform large‐scale variables to low‐dimensional (discrete) scale‐independent statistically weighted variables allowing for the selection of the most informative, robust, and reproducible categorical variables with the ability to stratify patient survival risk. In this study, we used an advanced version of the previously published software and algorithm [37, 43]. The following is a general description of how patient stratification in the risk groups can be utilized as a measure of survival prediction and as a method of selecting survival predictors (genes) for multivariate prognostic model.

#### 2.3.1 1D‐Data Driven grouping (1D‐DDg)

Assume a gene expression data set with *i* = 1, 2, …, *N* genes whose intensities are measured for *k* = 1, 2, …, *K* patients. The log‐transformed intensities of gene *i* and patient *k* are denoted as *y_i,k_*. Associated with each patient are a clinical outcome continuous data (e.g., survival time) and a nominal (yes/no) clinical event (e.g., tumor recurrence). Assuming that *K* clinical outcomes are negatively correlated with the vector of expression signal intensity *y_i_* of gene *i*, patient *k* can be assigned to the high‐risk or the low‐risk group according to

(1) $$x_{k}^{i}=\left\{\begin{array}{c} 1\left(\text{high risk}\right),\text{if}\;y_{i,k}>c^{i},\\ 2\left(\text{low risk}\right),\;\text{if}\;y_{i,k}<c^{i}, \end{array}\right.$$

where *c^i^* denotes the predefined cutoff of the *i*th gene’s intensity level. The clinical outcomes or events are subsequently fitted to the patients’ groups by the Cox proportional hazard regression model [46]:

(2) $$\log h_{k}^{i}\left(t_{k}¦x_{k}^{i},\beta_{i}\right)=\alpha_{i}\left(t_{k}\right)+\beta_{i}\cdot x_{k}^{i},$$

where $h_{k}^{i}$ is the hazard function and $\alpha_{i}\left(t_{k}\right)=\log h_{0}^{i}\left(t_{k}\right)$ represents the unspecified log‐baseline hazard function; β is the 1 × *N* regression parameters vector; and *t*_k_ is the patients’ survival time. To assess the ability of each gene to discriminate the patients into two distinct genetic classes [defined by Eqn (1)], the Wald statistic (W) [46] of the β*_i_* coefficient of the model Eqn (2) is estimated by using the univariate Cox partial likelihood function [47], estimated for each gene *i* as

(3) $$L\left(\beta_{i}\right)=\prod_{k=1}^{K}{\left\{\frac{\exp\left(\beta_{i}^{T}x_{k}^{i}\right)}{\sum_{j\in R\left(t_{k}\right)}\exp\left(\beta_{i}^{T}x_{j}^{i}\right)}\right\}}^{e_{k}},$$

where *R* (*t_k_*) = {j : *t_j_* ≥ *t_k_*} is the risk set at the time *t_k_* and *e_k_* is the clinical event at the time *t_k_*. The actual fitting of the model Eqns ((2), (3)) is conducted by the survival package in *R* (https://cran.r‐project.org/web/packages/survival/index.html). The genes with the largest β*_i_* Wald statistics are assumed to have better group discrimination ability and thus called survival significant genes. These genes are selected for further confirmatory analysis or inclusion in a prospective gene signature set. Note that log‐rank statistics were also included in our algorithm and shown similar or in some cases slightly better *P*‐value.

Note, how the stratification of patients in Eqn (1) depends on predefined cut‐off values (*c^i^*). In most real‐world scenarios, such values are not known in advance. Our 1D‐DDg method builds on the described workflow, by identifying the ideal cutoff without needing any prior information. First, for each gene *i*, we compute the tenth quantile (*q* ^i^_10_) and the 90th quantile (*q* ^i^_90_) of the distribution of K* signal intensity values. For every value, the algorithm performs the splitting of patients (1), fits the clinical event to the patient groups Eqn (2), and finally calculates the Wald statistic of β*_i_* Eqn (3). In other words, within (*q* ^i^_10_, *q* ^i^_90_), we search for the value* which corresponds to the minimum $\beta_{i}^{z}$ *P*‐value (here *z* = 1, …, *Q*) and that most successfully discriminates the two unknown risk groups.

We note that at the time of patient stratification we cannot tell which group is associated with higher or lower risk. The 1D‐DDg method predicts risk by analyzing the survival times of the groups. The group with lower mean survival times will be classified as ‘higher risk’, while the group with higher mean survival will be labeled as ‘lower risk’. According to this classification, two possible relationships exist between patient risk (lower risk, higher risk) and the expression pattern of a given gene (higher expressed, lower expressed). In the case of a parallel pattern, ‘higher risk – higher expression’ or ‘low risk – low expression’, the relatively higher prognostic gene expression level is associated with the poorer prognosis (a gene exhibits pro‐oncogenic behavior). In the case of antiparallel pattern ‘higher risk – low expression’ or ‘low risk – high expression’, the relatively higher prognostic gene expression level is associated with better prognosis (a gene exhibits tumor suppressor‐like behavior).

In our current work, the Rosetta and MetaData cohort datasets were used that contain both expression microarray data and the corresponding clinical information. Our survival prediction analysis was focused on the identification of the shortlist of survival significant genes of the *WAVE* complex, *RAC1* and *NDEL1*, all of which encode proteins constituting or interacting with the WAVE complex. Input list of the genes includes genes *WASF(1,2,3)*, *ABI* (1/2/3), *CYFIP(1*,*2)*, *NCKAP1*, *BRK1*, *RAC1*, and *NDEL1*, represented by the probe and probe sets localized in the 3’UTR of the selected genes on both microarray platforms.

The mRNA expression profiles of the selected genes are considered as putative predictors of the disease outcome. The 1D‐DDg analyzed the survival prediction property of the Rosetta and Metadata expression microarray signals corresponding to WAVE complex members and also *RAC1* and *NDEL1* as independent variables. An expression signal, called prognostic variable, is selected for further analysis if in both cohorts the DDg cutoff value(s) provide discrimination of the patients onto survival risk groups at *P* ≤ 0.05. To keep a reasonable compromise between sample size, the imbalance of distinct risk groups in a patient cohort, reproducibility across the different cohort and prognostic significance of the putative prognostic variables selection step, we were also allowed to include in the prognostic variables set up to two variables, if for a given variable in one dataset (e.g., DFS, Metadata) *P* ≤ 0.15. Thus, the output of 1D‐DDg analysis for Metadata or Rosetta cohorts includes the same list of reproducible prognostic variables (gene IDs) defined by the same gene lists, a similar survival prediction pattern of the identical variable (gene ID), cohort‐specific gene expression cutoff values dichotomizing the patients on to relatively low‐risk (code 1) and high‐risk (code 2) groups [37, 43, 44, 45].

Next, using the results of 1D‐DDg, our two‐dimensional grouping (2D‐DDg) method, and statistically weighted voting grouping (SWVg) we constructed the robust and synergistic multigene prognostic signature. The ability of individual prognostic variables (voting weight) to stratify patients in risk groups is represented by the *P*‐values associated with log‐rank statistics.

#### 2.3.2 Statistically Weighted Voting grouping (SWVg)

SWVg is an automatic method of prognostic feature selection and disease risk prediction that allows the construction of an optimized, multivariable, prognostic classifier [37]. The input data were provided by the 1D‐DDg method. The ability of individual prognostic variables to stratify patients is represented by the *P*‐values associated with the Wald statistic (calculated in the 1D‐DDg). These *P*‐values are used to calculate the relative weight of individual variables in the multivariable classifier. This information is used to construct a decision rule and to assign a patient to one of the risk subgroups.

In practice, the list of genes is ordered in ascending order according to the *P*‐values generated from 1‐DDg. The weight *w_j_* is calculated by the formula

(4) $$w_{j}=\frac{‐\log\left(P_{j}\right)}{\sum_{m=1}^{N}\left(‐\log\left(P_{m}\right)\right)},$$

where *P*_j_ is the *P*‐value of gene *j* in the 1D‐DDg procedure. Then, the new numeric grouping value for sample *i* could be calculated by the formula

(5) $$G_{i}^{N}=\sum_{j=1}^{N}w_{j}G_{\mathrm{ij}},$$

where *N* is the number of genes and *G*_ij_ is the group allocation for sample *i* assigned by gene *j* in the 1D‐DDg. In the case that samples are divided into two groups, patient *i* could be separated into two groups (2 = ‘high‐risk’, 1 = ‘low‐risk’) at a predefined cutoff value (*G*_c_) of $G_{i}^{N}$ with the following:

(6) $$y_{i}^{N}=\left\{\begin{array}{c} 1\left(\text{high}-\text{risk}\right),\text{if}G_{i}^{N}>G_{C}\\ \;0\left({\text{low}}_{\text{risk}}\right),\text{if}G_{i}^{N}\leq G_{C} \end{array}\right.$$

A Cox proportional hazard regression model is estimated by using a univariate Cox partial likelihood function with the method described in the 1D‐DDg procedure. Wald statistic of ${\hat{\beta }}^{j}$ is estimated and serves as an indicator to evaluate the ability of group discrimination for gene *j* at cutoff *G*_c_. The searching space of *G*_c_ is from 0.2 to 0.8, with an increment of 0.01 for each step. The *G*_c_ that provides the minimum log‐rank *P*‐values in the searching space is the optimized *G*_c_. The above‐described procedure is repeated for different *N*, which varies from 3 to the number of genes assigned. The number (*N*_opt_) and combination of genes are optimized for minimum log‐rank *P*‐values.

A similar procedure is applied when the samples are divided into three groups. Two cutoff values (*G*_c1_, *G*_c2_, *G*_c1_ < *G*_c2_) of $F_{i}^{N}$ selected and then used to calculate the grouping variable according to the following formula**:

(7) $$y_{i}^{N}=\left\{\begin{array}{cc} 1 & \;\left(\text{low risk}\right)\text{if}\;G_{i}^{N}>G_{\text{C2}}\\ 2 & \left(\text{intermediate risk}\right)\;\text{if}\;G_{C1}<G_{i}^{N}\leq G_{\text{C2}}\\ 3 & \;\left(\text{high risk}\right)\text{if}\;G_{i}^{N}\leq G_{\text{C1}} \end{array}\right.$$

A Cox proportional hazard regression model and log‐rank statistic estimates are computed. *G*_c1_ in Eqn (7) is searched in the range of 0.2 and 0.44, with an increment of 0.01 for each step, while *G*_c2_ is searched in the range 0.56 and 0.8, with an increment of 0.01 for each step. *G*_c1_, *G*_c2_ are optimized for the minimum value of summation of pair‐wise log‐rank *P*‐values of three survival curves.

The most significant and robust cut‐off value does not always result in balanced groups (i.e., one group may only contain a few patients). In our work, we aim to define risk groups that the smallest group contains at least 10% of the total patients. In cases where ‘the best’ cut‐off value resulted in unbalanced groups, and other values could stratify patients with statistically significant Wald statistics, we opted to use these alternatives.

Note that before the execution of Eqn (7), we recode the group values of the high‐risk group from 2 to 0. Using the modified values to calculate $G_{i}^{N}$ in Eqn (5) will result in $G_{i}^{N}$ closer to 0 for patients with higher risk. Conversely, patients with lower risk will have $G_{i}^{N}$ closer to 1. As a result, patients for whom $G_{i}^{N}>G_{\text{C2}}$ is true will constitute the lower risk group. Patients with $G_{i}^{N}$ that is below *G*_c1_ will be in the high‐risk group. Patients whose $G_{i}^{N}$ falls between *G*_c1_ and *G*_c2_ are classified as moderate risk.

To construct the multivariate prognostic signature, the SVWg starts with paired gene expression data using the two‐dimensional grouping (2D‐DDg) method [37, 44, 45]. For the given two variables domain and the 1D‐DDg determined cutoff values of these variables, the 2D‐DDg identifies two mutually excluded subdomains in the 2D domain that maximize discrimination of all subjects (patients) onto low‐ and high‐risk groups. The possible distinct subdomain combinations in the 2D domain are called ‘designs/models’ of the patient’s grouping.

SWVg adds the next prognostic variable that increases differentiation between risks of the groups and allows a selection of a synergistic multivariable signature based on the summation of the statistically weighting variables in a stepwise multivariable fashion. Less stringent statistical criteria (weights) are used by SWVg when the next most significant prognostic variable is added to the survival prediction model. The sample size and data quality constraints are included in the algorithm allowing the SWVg to minimize the number of prognostic variables (predictors) and reduce the signature identification overfitting risk keeping high confidence and reproducible prognostic multivariate model.

The multivariate method starts with the most significant prognostic variable (1‐st rank predictor) paired with the next most significant predictor. These features in combination provide a synergistic effect, robust prognostic signature, and provide consistency between the signatures derived in the cohorts.

#### 2.3.3 Optimization of the 2D‐DDg method for correlated covariates (gene expression value pairs)

In many datasets, the gene pairs (*A* and *B)* expressions may be correlated positively or negatively due to some context regulatory mechanisms (interaction due to common medical condition(s) and similar treatment). The paired correlation analysis could be used to improve the significance and robustness of patient’s risk group stratification. In this section, we describe an extension of our 2D‐DDg method.

Let *N* denote the number of nonduplicated samples of the population (patient cohort). Let {*X*, *Y*} denote the *N* random variable (r.v.) pairs (e.g., gene expression levels in the *N* samples that associated with *N* patient survival data (event and time after disease diagnostics or last follow up)), where the expression levels *X* and *Y* of the genes *A* and *B*, respectively. If the correlation measure between r.v. *X* and *Y* significant, the DDg defined risk group separation cutoff value (gene expression value determined a patient to the given risk groups) of bivariate r.v., could be optimized due to the variable’s dependence. In such cases, we can define the ‘interaction effect’ (synergy) between *A* and *B* data into 2D‐DDg prediction analysis as follows.

The method calculates the Kendal tau (or Spearman) correlation coefficient between all possible paired of r.v., specifies significantly correlated pairs, and then parameterizes the linear regression model quantifying the stochastic association between two r.v.

(8) Y=α+βX+ϵ,

where *x* is the vector of gene *A* expression values, *x* = {*x*_1_, *x*_2_, … *x_N_*}; *y* is the vector of gene *B* expression values, *y* = {*y*_1_, *y*_2_, … *y_N_*}; ε represents an additive error term that may stand un‐modeled determinants or random statistical noise: ε = {ε_1_, ε_2_, … ε*_N_*} *N* is the number of samples; α and β are parameters of the linear regression model. α is a y‐intersect of the line and β is a slope of the line. We estimate the parameters using the least squares method. The estimated parameter values denote as $\hat{\alpha }$ and $\hat{\beta }$.

Using parameterized Eqn (8) for the vector component pair ($X,Y‐\hat{\alpha }$) defined in the form

(9) $$\left(y_{i}‐\hat{\alpha }\right)=\hat{\beta }x_{i},i=1,2,\ldots \;,N,$$

we calculated the shortest distance of a particular point Q (x,y) from the regression line. To do this, we use a rotation of orthogonal coordinate system formula of point Q {$x,y‐\hat{\alpha }$} as the following

(10) $${\bar{x}}_{i}=x_{i}\cos\gamma +\left(y_{i}‐\hat{\alpha }\right)\;\sin\gamma ,$$

(11) $${\bar{y}}_{i}=‐x_{i}\sin\gamma +\left(y_{i}‐\hat{\alpha }\right)\;\cos\gamma ,$$

Where $\left\{{\bar{x}}_{i},{\bar{y}}_{i}\right\}$ are the coordinates of point Q in the new orthogonal coordinate system rotated on the angle γ. Using trigonometric formula, $\hat{\beta }$ = tanγ, and we obtain.

(12) $${\bar{x}}_{i}=\left(x_{i}+\hat{\beta }\left(y_{i}‐\hat{\alpha }\right)\right)/{\left(1+{\hat{\beta }}^{2}\right)}^{1/2},$$

(13) $${\bar{y}}_{i}=\left(‐\hat{\beta }x_{i}+\left(y_{i}‐\hat{\alpha }\right)\right)/{\left(1+{\hat{\beta }}^{2}\right)}^{1/2},$$

Equations (12,13) are used in our study for the calculation of new coordinates of the objects and corrected cutoff values $C\left\{{\bar{x}}^{*},{\bar{y}}^{*}\right\}$ defined by DDg for prediction of the low‐ and high‐risk groups in the patient cohort.

We included and used our rotation of orthogonal coordinate system approach in the 2D‐DDg method to improve the significance of the patient's separation on the relatively low‐ and high‐risk groups. Our analysis showed that in the high‐correlated genes, this method improves the statistical significance of results obtained in DDg methods, but also could lead to more robust grouping and reproducibility of the risk model across distinct patient cohorts. For instance, in the case of *ABI1‐ BRIK1* pair of the Rosetta cohort, our standard 2D‐DDg survival prediction analysis of DMFS provided near the borderline statistical significance of patients grouping (*P* < 0.05). However, a strong positive correlation between expressions of these two genes was found (*P* < 0.0001), suggesting common coregulatory mechanisms.

#### 2.3.4 Prognostic models, correlations, and reproducibility of the ABI1‐based prognostic signature genes

According to our selection criteria of prognostic variables (Methods), 1D‐DDg selected 5 genes of WAVE complex (*ABI1*, *BRK1*, *CYFIP1*, *CYFIP2*, and *WAVE3*) and 2 genes (*RAC1* and *NDEL1*) encoding the proteins RAC1 and NADEL exhibit ‘interaction’ with WAVE complex components. Our 7 genes were representative be unique probe sets on Affymetrix U133 A&B and Rosetta microarray platforms (Table S1). Figs. S3,S4 and Table S2 show that across different microarray platforms DFS and DMFS survival patterns *ABI1*, *BRK1*, *CYFIP1*, and *RAC1* are commonly reproducible and classified as pro‐oncogenic, while *CYFIP2*, *NDEL1*, and *WAVE3* are mostly classified as tumor suppressor‐like genes. However, because the system of interactive molecules is open, stochastic, and nonlinear for some genes (e.g., *WAV3*), the variations of these prognostic properties (as a component of the system) could be unstable and expressed alternative functions.

Note that over data sets and event types (e.g., DFS, DMFS), the prognostic pattern of expression changes for some individual genes (e.g., *WASF3*) was in some cases not the same (classified as proto‐oncogene or tumor suppressor like). However, the pro‐oncogenic pattern (upregulated expression–poor prognosis) of *ABI1*, *BRK1*, and *RAC1* or the ‘tumor suppressor’ pattern (upregulated expression–good prognosis) of *NDEL1* and *CYFIP2* expression was highly reproducible between our datasets.

In the context of co‐expression, the METABRIC, Rosetta, and Metadata data, *ABI1* expression is positively correlated with the expression of *BRC1*, *CYFP1*, and *NDEL1*. It is also not correlated with *RAC1* expression and is negatively correlated with *CYFP2* expression.

### 2.4 Mouse primary tumors RNA‐seq

Gene expression profiles from primary breast tumors of PyVT heterozygous and homozygous mice with and with *Abi1* disruption were detected with the Illumina NextSeq platform (GSE162815). Two tumors from a single mouse from each of the four groups were sequenced. Two runs were performed on consecutive days for increased depth. Illumina’s breast cancerl2fastq program was used for the conversion of base calls to FASTQ files. This resulted in two read files due to the paired‐end sequencing protocol. STAR was used to align sequences to the GRCm38/mm10 mouse genome. STAR was also used for the quantification of reads per gene. Raw counts between tumors and days for each genotyped were summed for maximum depth. Fold change was calculated between *Abi1* wild‐type and *Abi1* knockout mice for each genotype.

### 2.5 Animals

Transgenic PyMT mice (JAX no. 022974; C57BL6) and mammary‐specific *Cre* mice (JAX no. 003553, Line D; mixed strain) were purchased from Jackson Laboratory. *Abi1*‐floxed mice were generated by the Kotula Laboratory [16] (MGI : 4950557; Abi1^tm1.1Lko^, C57BL6). Female PyMT mice with conditional *Abi1* knockout were generated by crossing PyMT transgenic males to homozygous Abi1 females to produce PyMT transgenic males heterozygous for Abi1 floxed allele (PyMT; Abi1 fl/wt). PyMT; Abi1(fl/wt) males were backcrossed to homozygous Abi1 females to produce PyMT; Abi1(fl/fl) males. In parallel, transgenic Cre animals were crossed with homozygous Abi1 animals to generate transgenic Cre animals heterozygous for Abi1 [MMTV‐Cre; Abi1(fl/wt)]. To generate experimental animals, male PyMT; Abi1(fl/fl) were crossed to female MMTV‐Cre; Abi1(fl/wt). All breeders used were at least 8 weeks of age. Genotyping was performed using ear snips (Transnetyx, Cordova, TN). As mammary glands were the tissue of interest, only female experimental animals were analyzed. Female animals were sacrificed at designated time points (5, 7, and 12 weeks, for developmental studies, *n* = 5 animals per genotype; or seven weekly time points starting with the tumor detection (at week 0, 1, 2, 3, 4, 5, and 6, *n* ≥ 6 mice), when tumors reached 2.0 cm, or when animals displayed signs of distress as per the guidelines of the National Research Council Committee on Recognition and Alleviation of Distress in Laboratory Animals. For primary and lung metastasis tumor studies, animals (*n* ≥ 6 mice) were sacrificed age between 17 and 26 weeks. All animals used in the studies described herein were housed in ventilated microisolator caging under HEPA‐controlled environmental conditions and maintained under the supervision of the SUNY UMU Institutional Animal Care and Use Committee (IACUC no. 393).

### 2.6 Tumor palpation and measurements

Starting at weaning age, all female PyMT animals were palpated and measured for tumors biweekly. Tumor measurements and volume calculations were performed as previously described [48]. Total tumor burden over time was calculated for each animal (*n* = 6/genotype) and was plotted against the time since primary breast tumor was detected by palpation.

### 2.7 Mathematical Models and Estimated parameters in analyses of primary tumor kinetics and pulmonary metastatic node’s size frequency distribution

We estimated parameters of the tumor volume kinetics using the exponential function

$f\left(t;a,b\right)=\left(a‐b\right)\;*\;\exp\left(\mathrm{ax}\right)$,

where *t* is time, parameter *a* is the rate of cell volume growth, and (*a* − *b*) is the initial tumor volume. sigmaplot‐13 software was used to perform nonlinear regression analysis and the results visualization.

### 2.8 Proliferative activity and histological parameters of the mouse primary tumors

Mouse mammary parenchyma less than (<20 weeks) or greater than 20 weeks (>20 weeks) of age were examined by a blinded pathologist. Histologic sections of healthy control, homozygous control, heterozygous control, homozygous *Abi1*(KO, −/−), and heterozygous *Abi1*(KO, −/+) breast parenchyma were compared. The murine grades were determined according to published histologic criteria [35]. The Ki‐67 index is expressed as percent positivity from 500 nuclei counted in areas of highest positivity. The comparative analysis was performed for each group of mice vs normal and corresponding negative contours (breast parenchyma) of heterozygous *Abi1* (KO, −/+) and homozygous *Abi1* (KO, −/−) samples. Unpaired nonparametric Mann–Whitney U‐test was performed at *P* < 0.05.

### 2.9 Lung metastasis quantification

To quantify the metastatic area throughout the lung tissue, three 5μm sections from formalin‐fixed paraffin‐embedded mouse lungs (sectioned every 50 μm) were collected from each group (*n* ≥ 6 animals per genotype), stained with hematoxylin and eosin and imaged using an Omnyx digital pathology scanner (GE Healthcare, Boston, MA, USA) [49]. Images were quantified for the total number of metastatic foci using imagej software (NIH) and subjected to statistical analyses.

### 2.10 Western blot analyses

Western blots were performed as previously described [16]. Blots were probed with the following primary antibodies: ABI1 (Rockland, Pottstown, PA, USA; 1 : 1000), ABI2 (P‐20, Santa Cruz Biotechnology, Dallas, TX, USA; 1 : 500), ABI3 (GeneTex, Irvine CA, USA; 1 : 1000), WAVE1 (K91/36, MilliporeSigma, Burlington, MA, USA; 1 : 1000), WAVE2 (H‐110, Santa Cruz Biotechnology, Dallas, TX, USA; 1 : 1000), WAVE3 (W4642, Sigma‐Aldrich, St. Louis, MO, USA; 1 : 1000), or β‐actin (AC‐15, Sigma‐Aldrich; 1 : 10 000). Blots were incubated with SuperSignal West Pico or Femto ECL reagents (Thermo Fisher, Waltham, MA, USA) and imaged using a PxiTouch imaging system (SynGene, Bengaluru, Karnataka, India).

### 2.11 Immunohistochemistry, histology, and whole mount analysis

Immunohistochemical staining was performed with antigen retrieval following standard protocols. Tissue sections of normal mammary tissue were stained with anti‐CK8 (TROMA‐I, DSHB, Iowa, 1.1000) and anti‐CK14 (PRB‐155P, Covance, 1.250). Tumor sections (≥ 3 animals/genotype) were stained with the following antibodies : ABI2 (P‐20, Santa Cruz Biotechnology, 1.250), WAVE1 (K91/36, Millipore, 1.250), WAVE2 (H‐110, Santa Cruz Biotechnology, 1.250), and WAVE3 (Abreast canceram ab110739, 1.100). Stained sections were mounted on coverslips using Cytoseal XYL (Fisher) and imaged using a Nikon Eclipse Ci‐L upright microscope. Formalin‐fixed tumor specimens were stained with hematoxylin and eosin for histopathologic review. Grading of murine tumors was performed according to Fluck and Schaffhausen’s review of the model pathology [35]. Briefly, tumors were assigned a score of 0 (normal breast parenchyma), 1 (mammary hyperplasia consisting of dense lobules), 2 (mammary intraepithelial neoplasia; the murine correlate of ductal carcinoma *in situ*), 3 (early carcinoma characterized by early stromal invasion), or 4 (late carcinoma). The mitotic rate was determined by counting the number of mitotic cells in 10 high‐power fields (hpf). The mitotic rate was calculated for the areas of the tumor with the highest grade. Tumor sections were also stained with Ki‐67, with nuclei in cells in the highest grade areas counted to determine expression, which was reported as the percentage of positivity.

For whole mount staining, mammary glands were processed as previously described [50]. Stained whole‐mounted tissues were imaged using a Nikon D610 camera, and images were subjected to morphometry using ImageJ software (NIH). Terminal end buds, ductal length, and ductal branching were quantified as previously described [51, 52].

### 2.12 Statistics

Each cellular or biochemical experiment had technical (*n* ≥ 3) and biological (*n* ≥ 3) repeats. To determine statistically significant differences involving more than 2 biological groups, we used 1‐way and 2‐way ANOVA followed by *t*‐test, nonparametric tests, generalized univariate and multivariate linear models, correlations other analyses as stated elsewhere in the manuscript using Statistica 13, StatSoft); *P*‐value less than 0.05 was considered significant. Categorical data analyses were carried out using Sytel Studio‐9 software (Sytel Inc. Pume). Kinetic analysis and nonlinear models parameterization were done using SigmaPlot‐13 (systat Software takes) software.

### 2.13 Ethics approval and consent to participate

All animal studies were performed according to guidelines approved by the Institutional Animal Care and Use Committee of SUNY Upstate Medical University (Protocol no. 393). Publicly available datasets were used for all patient‐associated bioinformatics analyses in this manuscript.

## 3 Results

### 3.1 Upregulation of ABI1 gene expression in primary breast cancers correlates with aggressive, basal‐like phenotype and metastatic predisposition

**Fig. 1:**
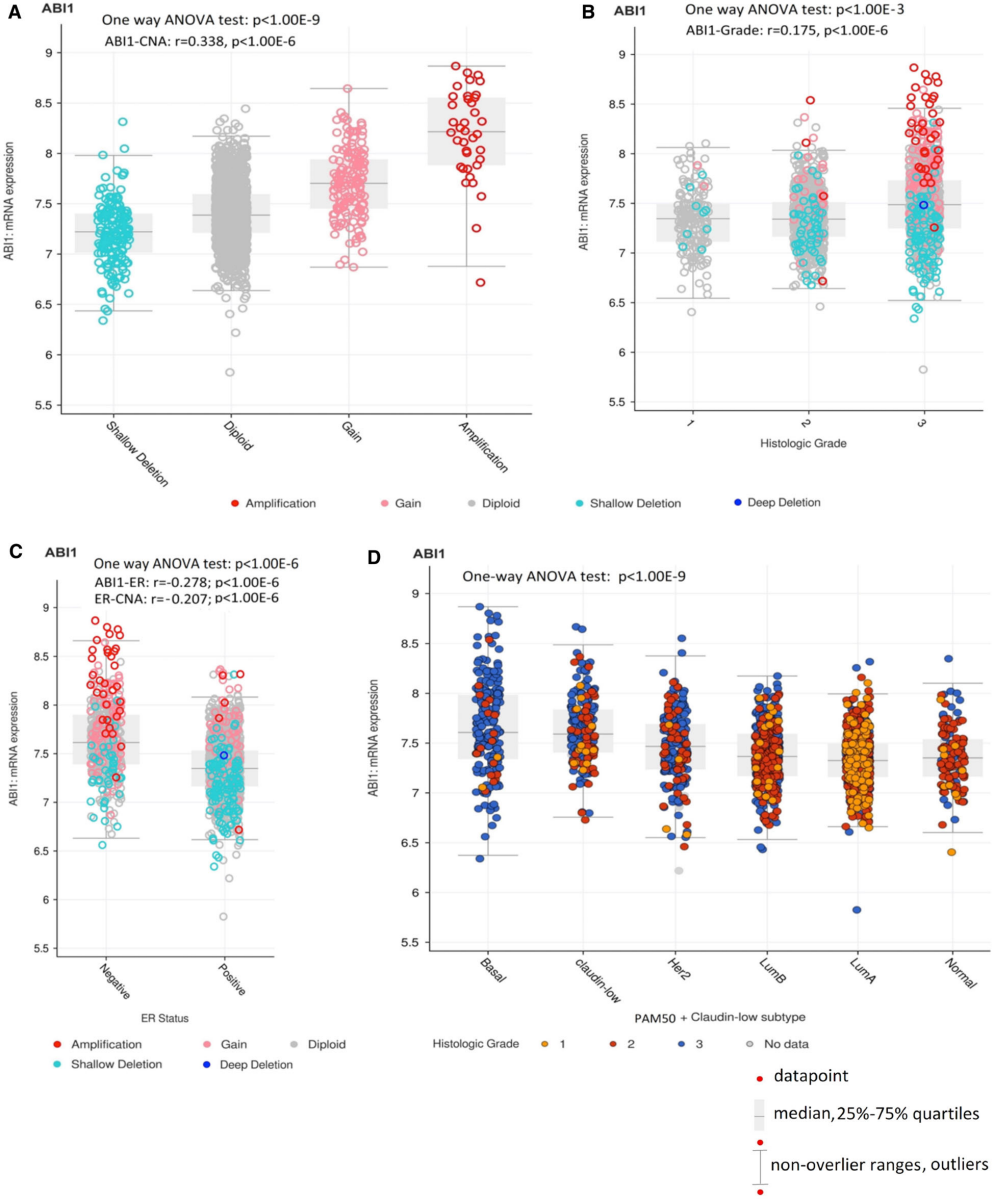
*ABI1* expression alteration is associated with copy number alteration (CNA) and high‐aggressive basal‐like breast cancer. Box Plots: (A) Putative *ABI1* DNA copy number alteration (CNA) drives *ABI1* transcription level in subpopulations of primary breast cancer patients [42]. The gene expression, CNA, tumor samples, and clinical datasets representing 1904 primary breast cancer samples were downloaded from METABRIC dataset (https://www.cbioportal.org/). CNA categorization is the following: shallow deletion: 1 (*n* = 166), diploid: 2 (*n* = 1554), gain: 3 (146), and amplification: 4 (*n* = 38). One‐way ANOVA test (Statistica 13) showed significant differences in the *ABI1* expression between the groups and also in the entire cohort (*P* < 1.00E‐9). Furthermore, the transcription level of *ABI1* is highly significant and positively correlated with CNA ((*r* = 0.338; *P* < 1.00E‐6; estimated by Spearman). (B) *ABI1* transcription level positively correlated with histologic grades (univariate and bivariate linear regression models testing shown significance at *P* < 1.00E‐6), however (C) negatively correlated with ER status. Bivariate linear regression models (Statistica 13) showed that both expression *ABI1* expression level and CNA are significant (*r* = −0.278; *P* < 1.0.00E‐6 and *r* = −0.207, *P* < 1.00E‐6 respectively); however, the *ABI1* expression provides a major contribution in the bivariate linear regression function). Correlate coefficients in (B) and (C) were calculated by Kendall. (D) *ABI1* overexpression is associated with basal‐like and claudin‐low breast cancer subtypes and aggressiveness of breast cancer scoring also by histologic grade. PAM50 (Basal‐like, HER2(+), luminal B, luminal A, normal‐like), and claudin‐low subtypes were ranked‐order according to the trend of decreasing of *ABI1* expression. One‐way ANOVA test (Statistica 13) showed significant differences in the *ABI1* expression between basal‐like, claudin‐low subtypes and other subtypes (*P* < 1.00E‐6). *ABI1* expression in the HER2 subtype was significantly higher than in luminal B or luminal A tumor subtypes (*P* < 1.00E‐6) and higher but less significant than in the normal‐like tumor subtype (*P* = 001). A negative trend in the *ABI1* expression across rank‐ordered tumor subtypes was mostly defined by relative overexpression of Basal‐like and claudin‐low tumor subtypes; it was highly significant (one‐way ANOVA; *P* < 1.00E‐9; Statistica 13).

ABI1 is an essential part of the WAVE regulatory complex, a major promoter of actin filament nucleation is often exploited by invasive tumor cells [53]. To elucidate the significance of the ABI1 in the pathobiology of human breast cancer, we carried out a retrospective analysis of METABRIC data of 1904 breast cancer patients (Fig. 1). We found that expression of ABI1 in primary tumors is strongly associated with copy number alterations (CNA) (Fig. 1A), overexpression with histologic grade 3 (Fig. 1B). There was a significant negative correlation ABI1 mRNA as well as ABI1 CNA with ER(+) status (Fig. 1C) and no correlation with the lymph node (LN) status of the patients (Fig. S5).

Moreover, ABI1 overexpression is associated with highly aggressive (grade 3) basal‐like and claudin‐low breast cancer subtypes (Fig. 1D). Additionally, using cBioPortal for Cancer Genomics tools (https://www.cbioportal.org/), we observed that high‐expressed and gained and amplified CNA *ABI1* are significantly enriched in the high genome instability integrative cluster 10 [42]. The cluster 10 molecular subtype is enriched by basal‐like cancer subtype tumors and clinically defined as triple‐negative, highly aggressive, drug resistance, and high‐risk metastasis tumor genes that includes numerous signaling molecules, transcription factors, mitotic, and other cell division genes associated in trans with this deletion event in the basal cancers, including alterations in *AURKB*, *BCL2*, *BUB1*, *FOXM1*, *KIF2C*, *KIFC1*, *RAD51AP1*, *TTK*, and *UBE2C*. Notably, many of these molecules are included genetic grade and poor survival outcome signatures [40, 42, 54, 55]. For instance, TTK (MPS1), a dual‐specificity kinase that assists AURKB in chromosome alignment during mitosis and promotes aneuploidy in breast cancer [42].

Thus, ABI1 expression shows strong positive correlates with histologic grading, negative correlation with ER status, and represents correctly the known ranked‐order of breast cancer subtypes according to their genetic grading classification (Fig. 1A–D; [40, 42, 52, 54, 55]). These findings allow us to consider ABI1 transcription level as a functional score of indicating (a) this gene locus instability, (b) ER(‐) status of the primary tumor, (c) histologic grading system estimator, and iv) a genetic variable that represents correctly known ranked‐order of breast cancer subtypes that reflect genetic grading and drug sensitivity/resistance of the tumor subtypes/groups.

Additionally, multivariate testing ABA1 expression variation as a random function of CNA, ER status, and tumor subtypes showed that CNA in basal‐like tumor subtype samples provides a major explanatory contribution of *ABI1* expression variation in our data (*P* < 1.00E‐6; two‐way ANOVA, Statistica 13).

### 3.2 Survival prediction analysis identifies *ABI1* as breast cancer metastasis prognostic marker and an important component of the multigene metastasis prognostic signature

We analyzed associations of survival data with microarray gene expression profiles of well‐established publicly available breast cancer datasets 39, 40, 41. These datasets were used to construct our Metadata and Rosetta microarray datasets (Methods, Supplementary Methods).

Firstly, we focused on the identification of the role of ABI1 expression in breast cancer survival associated with cancer progression/recurrence (defined DFS time) and metastatic process (DMFS time). Table S1 provides *ABI1* annotation and unique probe sets on Affymetrix U133 A&B and Rosetta microarray platforms utilized in our analysis. For stratification of the patients onto risk groups, we utilized 1D‐DDg, which approximates patient risks by analyzing the survival time functions of two (or more) patient groups given by the prognostic variable cutoff value(s) estimated statistically in a given patient cohort (Methods, Supplementary Methods). The examples of implementation of 1D‐DDg results for Rosetta and Metadata cohorts are presented in Figs. S3 and S4. Each figure shows the gene panels of two K‐M plots of disease‐free survival (DFS) (Fig. S3) and distant metastasis‐free survival (DMFS) (Fig. S4), respectively. The groups of the patients assigned to relatively low‐risk (step function line indicated by black color) and high‐risk (step function line indicated by red color) K‐M survival functions are defined by the gene expression cutoff value calculated by 1D‐DDg. The group with higher mean survival time is labeled as ‘low risk’, while the group with lower mean survival time is labeled as ‘high risk’. According to this classification, two possible relationships exist for the patients with lower and higher risks and the expression pattern of a given gene (higher expressed, lower expressed). In the case of a parallel pattern, ‘higher risk – the higher the expression’ or ‘low risk – the lower the expression’, the relatively higher prognostic gene expression level is associated with the poorer prognosis (a gene exhibits pro‐oncogenic behavior). In the case of antiparallel pattern ‘higher risk – the lower the expression’ or ‘lower risk – the higher the expression’, the relatively higher prognostic gene expression level is associated with better prognosis (a gene exhibits tumor‐suppressive‐like behavior).

Importantly, the prognostic association ‘higher risk – the higher the expression’ of ABI1 was statistically significant and reproducible over breast cancer cohorts (Figs. S3–S4). These results consist of our 1D‐DDg analysis of the gene expression of the *ABI1* gene and ABI1 protein in breast cancer patients found in RNA‐seq and proteomics databases (Fig. S6).

We propose that the high‐risk group of patients in our cohorts is associated with a higher frequency of metastatic events. Indeed, the metastasis event enrichment analysis (Table S3) showed that in both cohorts, the higher risk group was significantly enriched by metastatic events vs. the lower risk group. The fold change (FC) enrichment of metastatic event and *P*‐value was calculated using the exact test of two binomial distributions that showed FC = 1.38, *P* = 0.05 in Rosetta and FC = 1.96, *P* = 0.033 in Metadata dataset, respectively.

Next, we used the results generated by the 1D‐DDg survival prediction method, which automatically selects survival significant prognostic variables (survival significant genes represented by microarray probes), as the input data for the 2D‐DDg [29, 32] that identifies the interaction effect between paired prognostic variables (gene pairs) [29, 32]. Fig. S7 shows the result of the implementation of 2D‐DDg survival prediction to Rosetta data (DFS and DMFS, respectively). These results show that in most gene pairs ABI1 improves the balance between risk groups and in some cases the bivariate partition of the patients provides more confident risk group differentiation. Similar results were observed for the Metadata data set (not shown).

Interestingly, in both our cohorts, *ABI1* expression is positively correlated with the expression of *BRK1*, *CYFP1*, and *NDEL1*, but is not significantly correlated with the expression of *CYFP2*, *WASF3*, and *RAC1* (*P* < 0.05, Spearman). These findings in most cases consist of the correlation analysis of *ABI1* and other gene expression from the METABRIC datasets (Table S4). Note *WAVE3*, *CYFIP1* prognostic models may be more data variation‐ and noise‐sensitive.

**Fig. 2:**
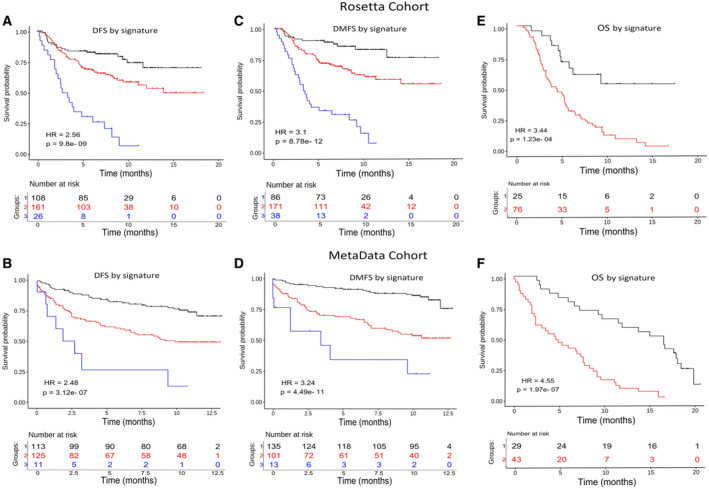
*ABI1*‐based prognostic signature predicts disease‐free and metastatic‐free survival risks. The disease‐free survival (DFS) and disease metastasis‐free survival (DMFS) of patients stratified based on the *ABI1*‐associated signature derived by our survival prognostic analysis method (see Methods for details) is shown using Kaplan–Meier survival curves for Rosetta (A, C) and MetaData cohorts (B, D). The Wald statistic *P*‐value and hazard ratio (HR) associated with the partitioning of the patients into distinct risk groups are also shown (see methods for details). Our method computationally categorizes each covariate (expression level of a gene) as a binarizing risk factor and stratifies each patient according to the multivariate expression pattern of the genes included in the signature (Table S1). In panels A, B, C, and D: black color line = ‘low‐risk’, red = ‘intermediate risk’, blue = ‘high‐risk’ groups. Panels E and F represent the overall survival (OS) time functions for the patients with metastasis detected after diagnostic and following surgical treatment. The black color line is associated with the group of patients with relatively better disease outcomes, while the red color is associated with patients with poor disease outcomes. The tables at the bottom of plots show the number of patients who survived in the predicted groups more than the given time point.

Finally, the SWVg algorithm was used to construct a survival group prediction model based on the combinations of 1DDg‐defined gene expression level models. Fig. 2A–D shows that in both Rosetta and Metadata cohorts, the method revealed a high confidence stratification of the patients onto three risk groups with high, intermediate, and low metastasis‐free survival time, called the ABI1‐based 7‐gene prognostic signature. Similar results were obtained for DFS time (results are not shown). Overall, the genes of the ABI1‐based 7‐gene prognostic signature provide robust functional associations and high‐confidence survival prediction properties.

### 3.3 ABI1‐based prognostic signature as a predictive tool for a metastatic event of breast cancers

Importantly, the ABI1‐based prognostic signature could serve as a predictive tool for a metastatic event of breast cancer patients. Indeed, Table S5 shows that in the case of DMFS the high‐ and intermediate‐risk groups are highly enriched for patients with metastatic breast cancer events compared to the low‐risk group (62% and 44% vs. 15% for Metadata and 79% and 34% vs 15% for Rosetta cohorts). The median and mean time values of metastatic events showed an inverse order in these risk groups. These findings suggest that our signature values defined in primary breast cancer samples can be used for the quantitative prediction of distant metastasis events and the time interval of metastatic event occurrence.

Using our ABI1‐based prognostic signature genes and specifying their expression cutoff values as done before, we were able to further stratify patients with metastatic events into relatively lower and higher OS time risk groups (Fig. 2E‐F). These results suggest that the *ABI1* and other genes of the prognostic signature are involved in the progression toward metastatic disease and may be mechanistic regulators of a subset of metastatic breast cancers.

To compare the prognostic significance, we used Rosetta and Metadata cohort's clinical and gene expression data and compared the ABI1‐based 7‐gene prognostic signature with commonly used clinical markers: estrogen receptor (ESR) and lymph node (LN) status (Fig. S5). While ESR status shows significant differences in DMFS in the survival of the Rosetta cohort (low vs. high expression) (Fig. S5A), it was not a predictive factor in the Metadata cohort (Fig. S5C). An opposite prognostic pattern was observed for LN status: It is not significant in the Rosetta cohort (Fig. S5B) but shows prognostic significance in the Metadata cohort (Fig. S5D). Additionally, univariate and multivariate analyses showed that LN and ER status is insufficient for reliable prediction of 3 risk groups (not shown).

Overall, the ABI1‐based prognostic signature provided robust, reproducible, and high confidence prediction models of DFS, DMFS, and OS (Figs. 1,2, Figs. S3‐S4, Tables S2,S5) and demonstrates high performance across different cohorts (Fig. 2; Table S2). Reproducibility of risk stratification of the patients with metastases in the Rosetta and Metadata datasets based on OS time supports this statement. Furthermore, the Abi1‐based signature predicts distant metastatic events more accurately than commonly used clinical factors (Table S6).

### 3.4 Loss of Abi1 does not grossly affect the long‐term development of normal mammary glands

While implicated in breast tumor progression, the role of ABI1 in normal mammary tissue remains unknown. To ensure that phenotypes that may be observed in our *Abi1* knockout (KO) breast tumor model result from the effects of ABI1 protein loss on tumor progression and not from an otherwise global effect on breast tissue, we conditionally deleted *Abi1* from mammary epithelial cells of non‐tumor‐bearing animals. As with most mammals, mouse mammary gland development occurs postnatally [56]. Mice are born with rudimentary mammary fat pads that develop into functional mammary glands upon the onset of puberty. Beginning at 5 weeks of age, the ductal tree begins to penetrate the mammary fat pad and continues until sexual maturation. This dynamic tissue reconstruction allows for examination of classical mammary structures such as ductal branches and terminal end buds (TEBs) (for an extensive review of mammary gland development, refer to Inman et al. [56]). Expression of the mammary‐specific CRE recombinase is under the control of the murine mammary tumor virus (MMTV) promoter and begins at ~ 21 days, allowing us to observe phenotypic changes in normal mammary gland tissue upon ABI1 loss [57].

**Fig. 3:**
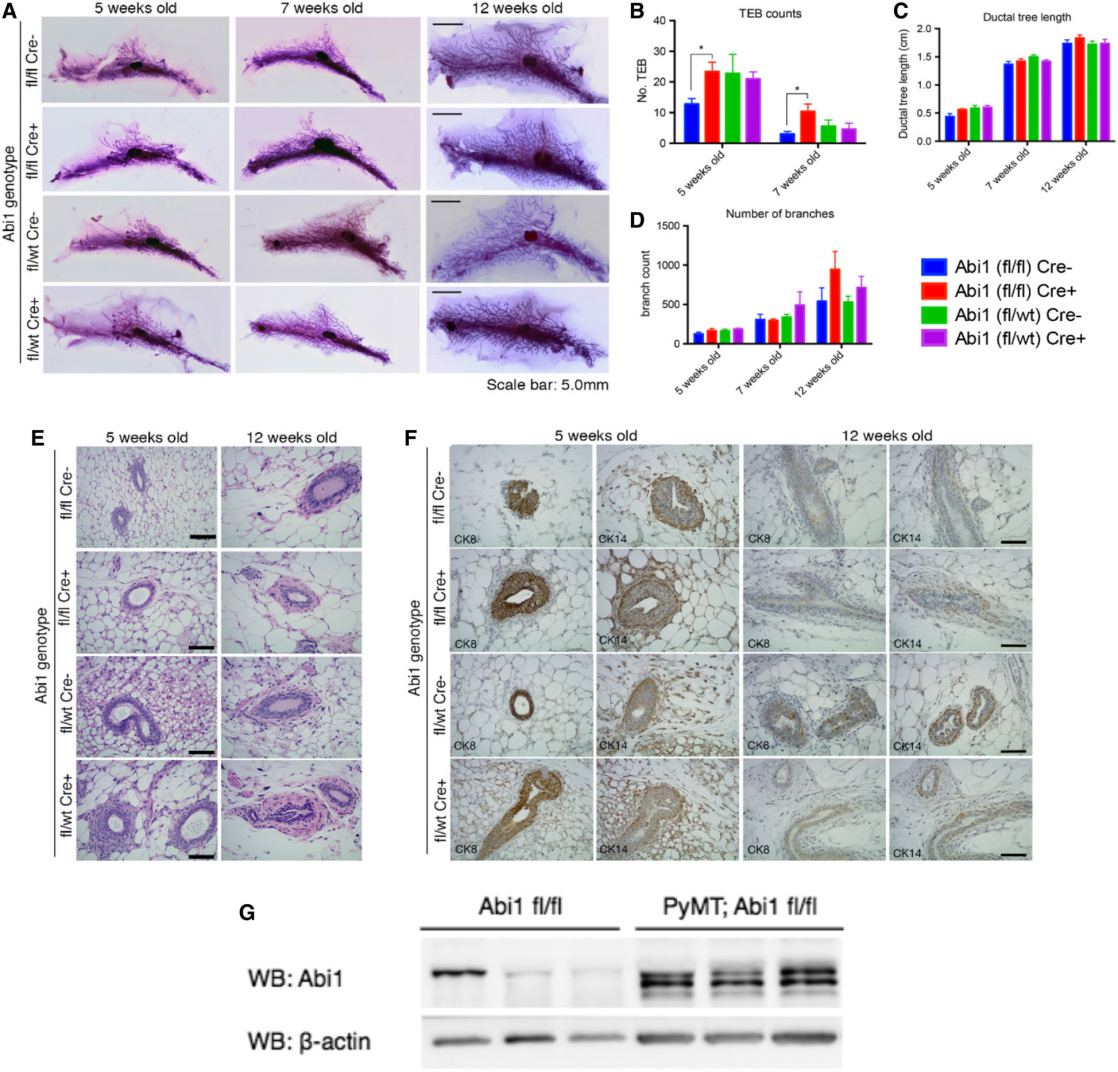
*Abi1* loss does not impact the long‐term development of healthy mouse mammary glands. (A) Whole‐mount analysis of the inguinal mammary gland stained with Carmine Alum reveals no gross changes in gland anatomy at 5, 7, or 12 weeks of age after CRE‐mediated deletion of *Abi1*. Morphometry of whole mounts reveals a significant increase in the number of terminal end buds in homozygous ABI1 null glands (B); however, this does not affect the elongation of the ductal tree (C) or the number of ductal branches (D). Scale bar, 5.0 mm. (E) Histological staining of mammary gland sections reveals no changes in tissue organization after CRE‐mediated loss of *Abi1*. Scale bar, 100 μm. (F) Immunostaining of mammary sections using markers for luminal epithelial cells (CK8) and myoepithelial cells (CK14) reveals sustained organization of the ductal epithelium in both control and ABI1 null mammary glands. Scale bar, 50 μm. Error bars indicate SEM. (* indicates *P* < 0.05, Student’s *t*‐test; *n* = 5 animals/genotype). (G) WB analysis indicates enhanced expression of Abi1 in mammary epithelium of Abi1(fl;fl) PyMT mice vs. Abi1 floxed mice Abi1 (fl;fl). Each lane represents one mammary gland (Abi1 fl;fl) or tumor [PyMT: Abi1(fl/fl)], (*n* = 3 mice).

To determine the effects of ABI1 loss on the development and structural integrity of normal mammary tissue, a whole mount analysis was performed on the inguinal mammary gland (see Materials and Methods). Gross examination revealed a modest impact of ABI1 loss on mammary gland development (Fig. 3A). We examined changes in the total number of terminal end buds (TEBs) as well as the number of ductal branches and total ductal tree length. TEBs are highly proliferative, tear‐shaped structures found at the distal end of the ductal tree that penetrate the mammary fat pad to facilitate ductal tree elongation and are involute upon completion of ductal tree extension [56]. Morphometry of mammary gland whole mounts showed a significant increase in the number of TEBs upon homozygous ablation of the *Abi1* gene and a trend toward increased branching, the latter of which did not reach significance (Fig. 3B,C); however, this does not seem to impact long‐term gland development, as ductal tree elongation remained unaffected (Fig. 3D). Heterozygous *Abi1* KO glands showed sustained TEB counts in 5‐ and 7‐week‐old whole mounts (Fig. 3B‐D).

In addition to dynamic tissue reorganization, mammary glands also have classically defined ductal structures. Murine mammary ducts are defined as lumens lined by an inner layer of luminal epithelial cells and an outer layer of myoepithelial cells [56]. Thus, analysis of this cellular organization would indicate whether there are organizational defects within the mammary duct upon *Abi1* deletion. Gross pathological examination of hematoxylin and eosin (H&E)‐stained mammary gland sections show the unaltered organization of epithelial cells and connective tissue within ducts (Fig. 3E). Immunohistochemical staining for cytokeratins 8 and 14, which mark myoepithelial and luminal epithelial cells, respectively, shows similar staining patterns in control and *Abi1*‐null mice (Fig. 3F) [58]. Taken together, we show that ABI1 loss does not affect the long‐term mammary gland development of healthy mice.

### 3.5 ABI1 protein level and gene dose regulate tumor growth in PyMT animals

*ABI1* overexpression has been implicated in promoting an aggressive breast cancer phenotype; however, its exact role in mammary tumor progression is still unclear 32, 33, 34. First, we established that PyMT transgene induces expression of *Abi1* in primary tumors vs. normal mammary gland epithelium of *Abi1* floxed mice (Fig. 3G); therefore, we concluded that PyMT mouse recapitulates overexpression of *ABI1* observed in human tissue, and thus, it is an appropriate model to examine the role of *ABI1* in breast cancer tumor progression. To determine efficiency of *Abi1* gene loss in our *Abi1* KO PyMT animals, we performed deep RNA‐seq analysis of representative primary tumors of each genotype (Table 1). We found that *Abi1* gene expression follows gene dosage effect as expected: 15.4‐fold in homozygotes and 2‐fold in heterozygotes vs. their respective controls. Several members of the WAVE complex were modestly downregulated or retained their expression in *Abi1* KO tumors vs. controls, while *Wave3* (*Wasf3*) was upregulated and *Cyfip2* was downregulated. An opposite effect on several WAVE complex genes expression in the heterozygous vs. homozygous animals was apparent (Table 1).

**Table 1:** Gene expression variability upon *Abi1* depletion in primary PyMT breast cancer tumors defined by RNA‐seq.

Mouse ID		G144	G164	G184	G174	Fold change depletion			
Genotype		fl/wt	fl/wt	fl/fl	fl/fl				
Cre		‐	+	‐	+	Heterozygous	Homozygous	Ratio	Treatment Effect^a^
Gene expression (RNA‐seq from primary tumors)	*Abi1*	14389	7343	15682	1018	**1.96**	**15.40**	**7.86**	**Yes**
	*Abi2*	8173	7189	8031	9719	1.14	0.83	0.73	No
	*Abi3*	146	271	343	195	**0.54**	**1.76**	**3.26**	**Yes**
	*Nckap1*	36435	34964	40591	38802	1.04	1.05	1.00	No
	*Wasf1*	88	66	85	118	1.33	0.72	0.54	No
	*Wasf2*	8563	10349	13894	11087	0.83	1.25	1.51	No
	*Wasf3*	261	151	95	221	**1.73**	**0.43**	**0.25**	**Yes**
	*Brk1*	10240	9103	11091	9920	1.12	1.12	0.99	No
	*Cyfip1*	23448	22528	31708	23030	1.04	1.38	1.32	No
	*Cyfip2*	521	1289	1633	580	**0.40**	**2.82**	**6.97**	**Yes**
	*Rac1*	25732	22621	27209	24431	1.14	1.11	0.98	No
	*Ndel1*	10923	10842	15082	13030	1.01	1.16	1.15	No

a Treatment effect is ‘positive’ (yes) if the fold change of gene expression for heterozygous and homozygous mice changed more than 1.5 times (bold text) in any direction and ‘negative’ (no) in other cases. RNA‐seq. expression profiles of WAVE complex, and Rac1 and Ndel1 genes (involved in WAVE complex stability and functionality) show the differences between heterozygous vs. homozygous Abi1 KO PyMT mammary tumors.

Interestingly, comparative analysis of the basal‐like vs. luminal breast cancer cell types markers in ABI1 KO mice showed that the genes of basal‐like cells (*Krt14*, *Vim*) are responded to *Abi1* depletion, however luminal cell type genes markers (*Krt8*, *Krt18*, *Sox9*, *Estr1*) do not (Table S7). The directionality of gene expression of *Krt14* and *Vim* in heterozygous and homozygous mice was different.

**Fig. 4:**
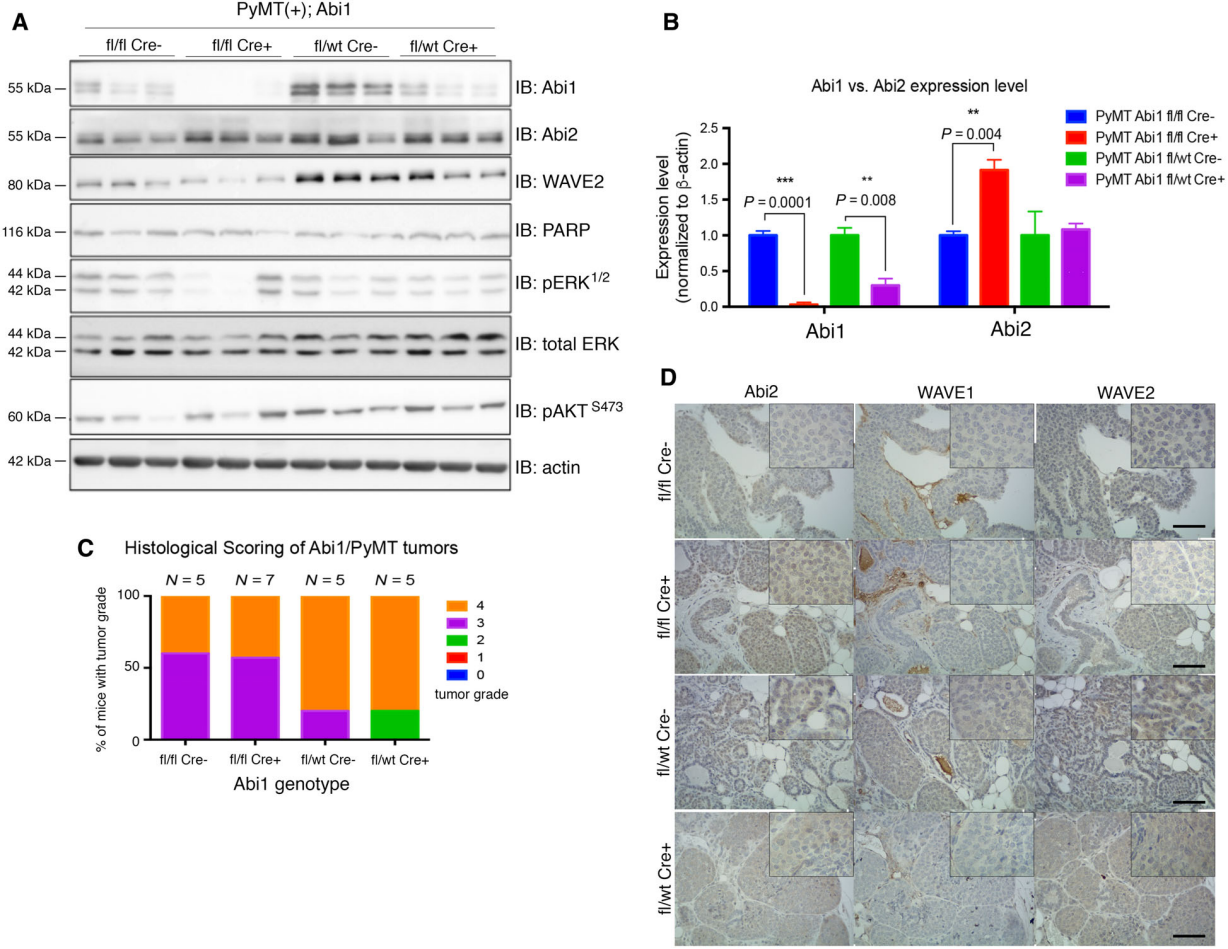
*Abi1* KO severely impacts WAVE complex gene expression dynamics. (A) Western blot analysis of primary mammary tumors from *Abi1* KO PyMT mice shows significant depletion of ABI1 protein, but only in the homozygote *Abi1* null is there significant upregulation of ABI2 protein as indicated by densitometry (B). Each lane represents one mammary tumor isolated from one animal of that genotype. Error bars indicate SEM. (*P* < 0.05, *t*‐test; *n* = 3 animals/genotype). (C) Analysis of primary tumor histology reveals no significant changes in tumor grade between controls and *Abi1* knockouts, (*P* > 0.05, *t*‐test; *n* ≥ 5 mice per genotype of age between 20 and 22 weeks were used for analysis, Table S8). Error bars indicate SEM). (D) Immunostaining with antibodies against WAVE complex proteins supports our findings that ABI2 is upregulated only in ABI1 null breast tumors. WAVE1 retained its low expression, while WAVE2 was concomitantly depleted with ABI1, in agreement with WB data, above. 20× magnification; inset, 40× magnification, Scale bar, 50 μm.

We have shown that *Abi1* KO mouse embryonic fibroblasts reliably show downregulation of WAVE2 [16]. Consistent with this finding, western blot analysis of *Abi1* KO breast tumors (tumor lysates from 3 mice/genotype) showed an appreciable reduction in WAVE2 expression in the absence of ABI1, recapitulating previously observed WAVE complex dynamics and dependence of complex stability on *Abi1* gene status (Fig. 4A) [16, 17]. Interestingly, WAVE2 expression remains relatively stable in heterozygous *Abi1* KO tumors, suggesting that a single copy of *Abi1* is enough to sustain WAVE complex stability to some degree, noting that there is still a noticeable loss in WAVE2 expression. Also, densitometric analysis of our western blots revealed significant upregulation of ABI2, another member of the ABI family, only in homozygous *Abi1* KO animals, in agreement with our previous findings (Fig. 4B) [16].

Based on our western blot findings, we next examined whether altered WAVE complex expression in the absence of ABI1 was recapitulated by immunohistochemical staining of tumor tissue (Fig. 4D). Similar to our western blot results, *Abi1*‐null tissue shows increased ABI2 expression in the cytosol, while WAVE2 shows moderate downregulation overall. WAVE1 is modestly expressed regardless of ABI1 status; therefore, it may not play a role in breast tumorigenesis in this model. Due to their ubiquitous expression, ABI1‐WAVE2 complexes are considered canonical WAVE complexes that drive F‐actin polymerization during cell processes [59]. As there is a concomitant loss of WAVE2 upon *Abi1* KO but sustained tumor growth in PyMT mammary tumors, it is possible that other factors contribute to ARP2/3‐mediated actin polymerization. Moreover, overall primary mammary tumor histopathology was not affected upon ABI1 loss (Fig. 4C; Table S8). While most of the primary tumors in either control or homozygous *Abi1* knockout animals remain in grades 3 or 4, some tumors in the heterozygous *Abi1* knockout appear to be in grade 2, further highlighting the impact of single copy *Abi1* deletion as opposed to homozygous deletion and suggesting other mechanisms may be induced in the complete genetic absence of *Abi1*.

### 3.6 *ABI1* gene dose regulates primary PyMT tumors growth kinetics

**Fig. 5:**
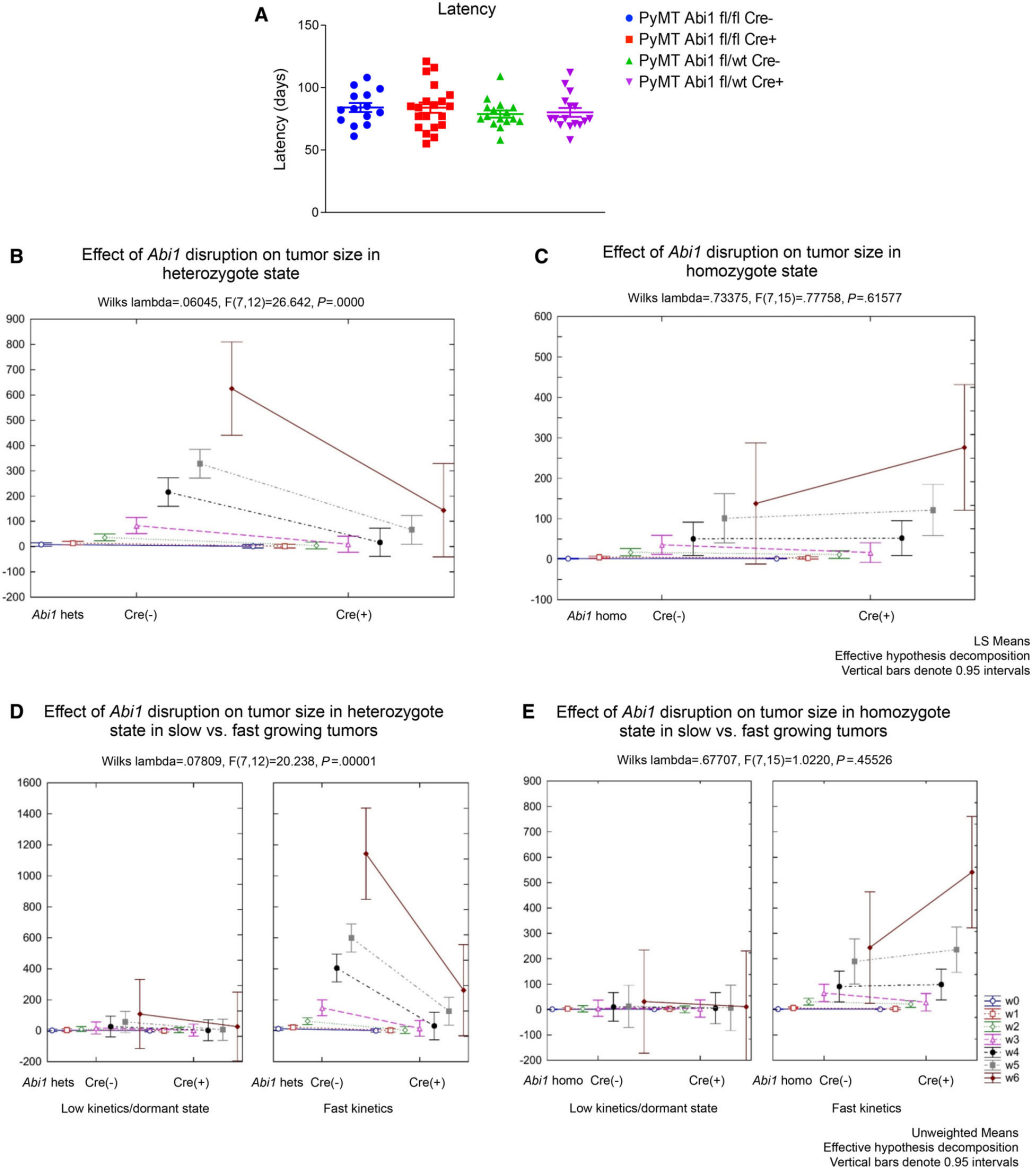
Primary tumor growth kinetics analysis indicates *Abi1* gene dose effect in heterozygous mice. (A) Primary tumor latency in PyMT animals is not significantly affected upon *Abi1* KO. The *X*‐axis of a panel (a) represents latency time comparison of the tumors in four treatment conditions defined on the upright corner of the panel (Abi1 fl/fl Cre‐, *n* = 14 mice; Abi1 fl/fl Cre+, *n* = 20 mice, Abi1 fl/wt Cre‐, *n* = 16; Abi1 fl/wt Cre+, *n* = 16 mice). (B‐E) Treatment effects of *Abi1* disruption (fw Cre(+) vs fw Cre(‐) and tumor kinetics of tumor size in heterozygous or homozygous miceGraphical tools of Statistica‐13 were used. Each plot on panels (B‐E) shows tumor size at seven‐time points (w0, w1, w3, w3, w4, w5, and w6 (see Methods)) (for Abi1 fl/fl Cre+, or Cre‐, *n* = 13 mice were used; for Abi1 fl/wt Cre+, or Cre‐, *n* = 11 mice were used). The line connects start (Cre(‐)) with the endpoint (Cre(+)) tumor size datasets allowing the comparison of tumor kinetic observations to be easily followed; mean values of tumor size are linked by direct lines at the same detection time point. Wilks lambda statistics and Fisher test were used for estimation of treatment significance. Panels (B) and (C) represent a visualization of the treatment effect (Cre(‐) v.s. Cre(+)) of *Abi1* on tumor size in observed time points. Vertical bars indicate 0.95 intervals, CI. An effective decomposition method of Statistica‐13 was used. The primary tumor size comparison in fastly growing mouse groups shows the exponential growth kinetics. (Methods, Table S10). To compare gene dosage effects within heterozygote and homozygote groups, mean values in 7 observed time points were compared (see Table S10 for details). Our results showed that in the cases of fast kinetics datasets, differences between the paired sample mean values were not significant for homozygote (*t*‐test, *P* > 0.15) but significant for heterozygote state (*t*‐test, *P* = 0.017). (See Methods and Table S10).

To determine the impact of *Abi1* disruption on mammary tumor initiation and progression, we used our *Abi1* KO mouse model to study the impact of *Abi1* loss on tumor progression and characteristics in the PyMT‐driven breast cancer. The PyMT model initiates spontaneous tumor formation with most mammary glands developing tumor nodes. Interestingly, KO mice *Abi1* do not significantly impact primary tumor latency (Fig. 5A). To determine the effects of *Abi1* expression on breast cancer progression, heterozygous and homozygous KO mice were used to study the growth kinetics (i.e., tumor volume changes over time) of sporadically occurring tumors. Tumor size was measured biweekly, starting from first day of tumor palpation. We collected and analyzed datasets from *Abi1* homozygous Cre(+) (*n* = 11), Abi1 heterozygous Cre(+) (*n* = 11), Abi1 homozygous Cre(‐) (*n* = 13), and Abi1 heterozygous Cre(‐) (*n* = 13) samples (Fig. 5B–E). The tumor kinetics showed two growth patterns. The analysis of tumor volume kinetic data in mice identified two tumor growth patterns, which are growing with very low or fast rates across all four genotypes (Fig. 5B–E; Tables S9). The fraction of tumors exhibiting the slower growth varied between 54% and 64% across the four experimental groups. Other breast tumor samples showed stable exponential growth with either moderate or high growth rates (Fig. 5B–E; Table S9). Using the one‐way ANOVA test, we found that in *Abi1* heterozygous KO model samples the tumor growth kinetics was strongly suppressed vs. control (Fig. 5D), while no significant effect was found in *Abi1* homozygous KO model tumor samples with some positive trend in the opposite direction in faster‐growing tumors (Fig. 5E).

### 3.7 ABI1 promotes the number and size of lung metastases in a gene dose‐dependent manner

**Fig. 6:**
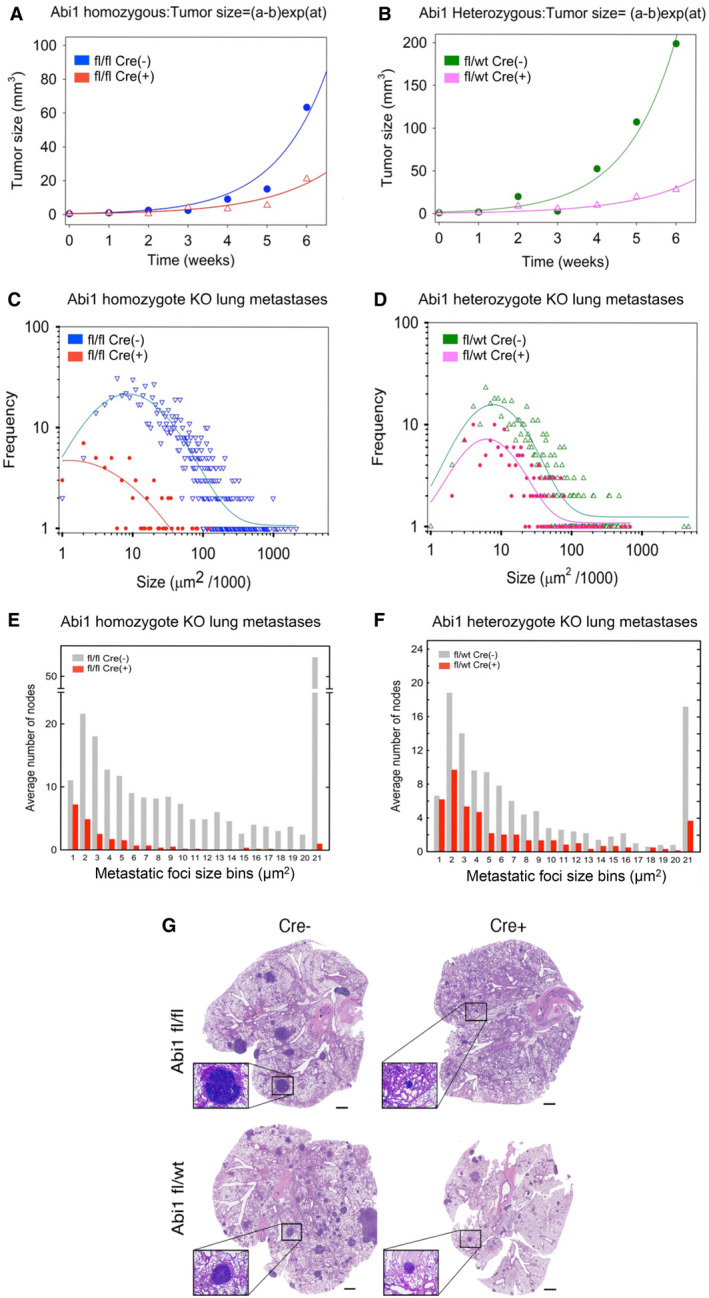
*Abi1* gene knockout reduces metastatic burden in heterozygous and homozygous mice. Representative tumor kinetics of primary (panels a‐b) vs metastatic tumors (panels c‐d). Panel (A) Comparison of the primary tumor volume kinetics in *Abi1* homozygous KO mouse (fl/fl; Cre+) (G209, data: red triangle; best‐fit function: red line) and the control *Abi1* (fl/fl Cre‐) mouse (G184, data: blue circle; best‐fit function: blue line). (B) Comparison of the primary tumor kinetics of *Abi1* KO heterozygous (fl/wt Cre+) mouse (G251, data: pink triangle; best‐fit function: pink line) and the *Abi1* control (fl/wt; Cre‐) mouse (G202, data: green circle; best‐fit function: green line). Kinetics of mean values (A and B) were fitted by exponential curve *f* (*t*; *a*, *b*) = (*a* − *b*) * exp(*at*), where *t* is time, constant *a* is the rate of cell population growth and constant (*a − b*) is the initial tumor population size. Each kinetic dataset includes seven time points (see also Table S10). The estimated parameters in *Abi1* fl/fl Cre (‐) tumors: *a* = 0.77 +/− 0.159, *t*‐test, *P* = 0.0047, *b* = 0.2 +/− 0.678, *t*‐test, *P* > 0.1 and in *Abi1* fl/fl Cre (+) *a* = 0.60 +/− 0.153, *t*‐test, *P* = 0.0039), *b* = 0.1 +/− 0.586, *t*‐test, *P* > 0.1. Estimated parameters in *Abi1* fl/wt Cre (‐) tumors: *a* = 0.79 +/− 0.091, *t*‐test, *P* = 0.001), *b* =−1.00 +/− 0.964, *t*‐test, *P* > 0.1, and in *Abi1* fl/wt Cre (+) *a* = 0.589 +/− 0.110, *t*‐test, *P* = 0.0031, *b* =−0.30 +/− 0.66, *t*‐test, *P* > 0.1. According to these results, differences between mean values of the tumor sizes in the studied groups in time are not significant. While primary tumor volume kinetics was not significantly different in these mice vs. their corresponding controls, (A, homozygous *ABI1* KO vs. control) and (B, heterozygous KO vs. control), the difference in metastatic tumor burden of the same mice within each mouse genotype was significant (C) and (D). Panels (C) and (D) show the frequency distributions of a lung metastatic foci size in the heterozygous and homozygous mice, which primary tumors kinetics showed on panels (A) and (B), respectively. Each Y‐axis value shown in the histograms (C‐D) represents a count of metastatic foci within a metastatic size normalized interval (a bin). The bin was defined by rounding the metastatic size divided by 1000 to the nearest integer, and the number of metastatic foci in each bin was counted. Based on our findings, the metastases size frequency distribution in the lung has skewed form with the long right tail. To provide a visualization of such frequency distribution, we used log_10_ − log_10_ plot. We used the same color for dots of the empirical distributions and the fitting function lines, as was indicated in the figures. Such empirical frequency distribution was modeled and parameterized using the shifted log‐normal distribution function: $f\left(x;\;y_{0},x_{0},a,b\right)=y_{0}+a\;*\;\exp\left(‐0.5\;*\;{\left(\ln\left(x/x_{0}\right)/b\right)}^{2}\right),$ where *x* is the node size and *y*_0_, *x*_0_, *a*, *b* unknown parameters. We estimated the parameters using the nonlinear curve fitting option of SigmaPlot‐13 software. Datasets and detailed results of the parameterization of this function are presented in Table S9. (E‐F) show histogram bar plots for the distribution of the average number of metastases foci size in the lungs of *Abi1* KO mice in comparison to their genetic controls. X‐axis indicates binning for every 5000 μm^2^ metastasis colony area size, with bin 1 representing 0–5000 μm^2^ and bin 21 representing 100001 μm^2^ and larger; Y‐axis: count of the samples within given binning interval (+/− SEM). The size stratification of individual metastatic colonies shows that mice lacking ABI1 still have relatively small metastatic colonies but they grow slowly or/and stay at dormant state and appear unable to establish macrometastases when compared to our controls (*P* < 0.001; Wilcoxon signed‐rank test). Lung metastasis quantification was performed following fixation, paraffin embedding and sectioning: three 5μm sections (sectioned every 50μm) were collected from each mouse (Abi1 fl/fl, Cre‐, *n* = 7; Abi1 fl/fl, Cre+, *n* = 6; Abi1 fl/wt, Cre‐, *n* = 6; Abi1 fl/wt, Cre+, *n* = 6; animals per genotype, age 18–22 weeks), were stained with hematoxylin and eosin, and imaged using Omnyx digital pathology scanner (GE Healthcare). Images were quantified using ImageJ software (NIH). Results of panels (E) and (F) support the results presented in (C) and (D). (G) Histological staining of representative lung sections reveals severely diminished metastasis upon deletion of the *Abi1* gene. Scale bar, 1 mm. Inset, 4× magnification.

Most *Abi1* KO PyMT mice demonstrated pulmonary metastasis within 6 months of the primary tumor detection. We noted that mice with fast‐growing tumors showed a positive trend for association with multiple metastatic events and large size metastatic foci in both Cre(‐) control groups (Fig. 6). To elucidate the role of *Abi1* gene dosage effect in lung metastasis, we first analyzed the tumor kinetic rates of the primary tumor growth vs. the largest tumor metastatic foci at 6 months within the same mice in Abi1 KO homozygous and heterozygous tumor groups (Fig. 6A–B). Fig. 5A–B shows a weak gene dose effect in both homo‐ and heterozygous primary tumor kinetics. To be more conclusive, we estimated parameters of the tumor volume kinetics using the exponential fit function *f* (*t*; *a*, *b*) = (*a* − *b*) * exp(*ax*), where parameter *a* is the rate of cell volume growth and (*a* − *b*) is the initial tumor volume.

No statistical differences between exponent rates in control and treatment were found (Materials and Methods). However, a comparison of the number and size of metastatic foci of *Abi1* KO animals indicated a strong gene dose effect (Fig. 6C–G). Fig. 6C–D shows the frequency distribution of pulmonary metastatic foci that exhibited the highest number of metastatic foci and largest metastasis size in the Abi1 fl/fl and fl/wt lung tissues. In each lung sample, the frequency distribution of the metastatic foci size shows the skewed form with long tails. We found that for each case, the frequency distribution of pulmonary metastatic foci size is fitted well by a discrete analog of shifted log‐normal distribution function (for better visualization the function approximated by continuous curves) (Table S9A–B and Methods). Estimated parameters of the distribution function we used to define significant differences between the shapes of the distribution functions shown in Fig. 6C–D (Table S9B). In particular, parameter *x*_0_ estimates a mode of the frequency distribution function which is most frequent size of micro‐metastasis foci. For Abi1 fl/fl Cre‐, fl/fwt Cre‐, fl/wt Cre+ data are variated between 6.3–8.6 µm^2^, but for fl/fl Cre+ focus size equals 1.3 µm^2^ (Table S9B). A comparison of *x*_0_ and the parameter *b* (basal (smallest) foci size at *x* = 0), Table S9B) of the best‐fit distribution function draw in Fig. 6C,D suggests a significant reduction of the multiple metastatic foci size and their numbers in the treatment cases fl/wt Cre+ and fl/fl Cre+. Additionally, statistical testing using the Wilcoxon signed‐rank method demonstrated significant differences between the observed frequency distributions of treatment v.s. control datasets (*P* < 0.0001). Comparison of the frequency distributions of the treatment groups provided a significant difference (reduction of median value in fl/fl Cre+ vs median value in fl/wt Cre+) (*P* < 0.0001). These results indicate a strong *Abi1* gene dose effect promoting lung metastases in both homozygous and heterozygous PyMT models but the effect in homozygous mice was stronger. Similar results were observed for pulmonary metastasis foci size bins (50 µm^2^) frequency distribution that includes all defined pulmonary metastatic foci datasets (Fig. 6E‐F). Representative lung tumor images are shown in Fig. 6G.

## 4 Discussion

Here, for the first time, we demonstrate the metastasis driver role of ABI1 in breast cancer tumor progression using the PyMT mouse model and clinical data from breast cancer patients. Our bioinformatics analyses revealed the significant role of human *ABI1* and a subset of the WAVE complex genes in the context of breast cancer progression and metastatic process.

In the Metadata and Rosetta cohorts, the high expression of *ABI1* demonstrated poor survival time patterns as indicated by survival time and is significantly associated with metastatic events. Moreover, in the large METABRIC cohort the *ABI1* expression is positively correlated with DNA CNA, histologic grade 3, and basal‐like phenotype, but negatively correlated with ER status and does not correlate with LN status. We identified the high confidence and reproducible multigene survival prognosis signature comprised of *ABI1* and six other genes: *BRK1, CYFIP1*, *CYFIP2*, and *WASF3*, which are the genes encoding WAVE complex members; and *RAC1* and *NDEL1* genes, which are upstream interactors and regulators of the WAVE complex [5, 14, 60]. Both RAC1 and NUDEL participate in the EMT pathway and play key roles in the metastatic migration of epithelial cells via the interaction with WAVE family proteins and the regulation of cancer‐determined pathways [5, 12, 61, 62, 63.

Collectively, our tumor progression and metastatic prognostic signatures allow for the identification of optimal gene expression cutoff values to stratify patients on low‐, moderate‐, and high‐risk subgroups based on DFS and DMFS times. Our survival prediction analyses establish the significance of *ABI1* gene expression as a pro‐oncogenic factor of primary tumor formation and metastasis in breast cancer patients. These findings support the experimentally testable working hypothesis that genetic mechanisms of ABI1 are key components in the metastatic breast cancer process.

Univariate and multivariate analyses and comparisons between Kaplan–Meier survival curves generated with our prognostic signature and those generated with either estrogen receptor (ESR) or lymph node status reveal that our signature outperforms these clinically used variables and could lead to better personalized and predictable treatment selection. This conclusion is supported by our co‐expression analysis between ABI1 and other members of the ABI1 survival (prediction) signature and the observed significant positive correlation between *ABI1* expression, CNA, histologic grades, and basal‐like phenotype vs. ER(+) luminal cancer phenotype—the clinical markers of aggressiveness, metastasis, and drug resistance frequency.

The availability of a genetically engineered conditional *Abi1* KO mouse permitted us to investigate the role of Abi1 downstream from the PyMT oncogene. By comparing the effects of one‐ and two‐allele inactivation of the *Abi1* gene, we were able to determine that ABI1 expression levels play an important pro‐oncogenic role in breast cancer tumor progression and metastatic disease. The two‐allele inactivation of the gene (in *Abi1* homozygote KO mice) and one‐allele inactivation of *Abi1* (in *Abi1* heterozygote KO mice) led to lower metastatic burden in the lungs.

Disruption of Abi1 in normal mammary epithelium led to a significant increase in terminal end buds at weeks 5 and 7 (Fig. 3B), but beyond that time point, the development of mammary glands was not affected (Fig. 3B–D). The increase in the TEB number, as well as the trend toward increased branching in tissue with Abi1‐disruption, warrants further investigation to determine whether ABI1 or other ABI proteins play a role in normal murine mammary gland development. To corroborate the findings of *Abi1*, the disruption of *Wasf3* gene also demonstrated no significant effect on mammary gland development [22]. *WASF3* is part of *ABI1* 7‐gene signature.

We observed that complete loss of ABI1 yields no difference in primary mammary tumor growth kinetics (Fig. 5C,E) and that lung metastasis is severely abrogated in both homozygous and heterozygous *Abi1* KO (Fig. 6C‐F). Thus, our findings strongly suggest that ABI1 is critical for pulmonary metastasis of aggressive breast tumors due to its essential role in sustaining WAVE complex dynamics. The WAVE complex is assembled from intimate interactions of five obligatory components: a WAVE, an ABI, a CYFIP, an NAP, and BRK protein, which are altogether products of 11 genes [6, 8, 9]. The study by Kirschner’s group demonstrated that the presence of all five WAVE complex proteins is required to form the functional WAVE complex *in vitro* [6]. Genetic inactivation of *Abi1* led to overall WAVE complex downregulation in MEF cells, but deregulation of individual WAVE complex proteins was also evident. These included the relative upregulation of ABI2. Similarly, upregulation of ABI2 is observed in breast tissue lacking ABI1 (Fig. 4A,B). Despite their homology and similarities in function, upregulation of ABI2 cannot sustain pulmonary metastases in homozygous *Abi1* KO animals (Fig. 6C,E,G), strongly indicating that ABI1 is critical for lung metastases in this model.

The lack of local effect on primary tumor growth in *ABI1* homozygous mice is difficult to explain in the context of the effect on lung metastases but raises the possibility for potential tumor suppressor role for *ABI1* in breast epithelial cells in some genetic contexts such as here downstream from the PyMT oncogene. ABI1 acts as tumor suppressor in several other tissues such as prostate [30].

Focus is a pathologic term describing cells that can grow as a colony and be seen only microscopically. In this study, we quantified differences in the number of multiple metastatic foci and the sizes of the breast cancer metastases. We found essential differences for both characteristics in the breast cancer metastases in the *ABI1* gene dosage‐dependent manner. Our experimental model results demonstrate the important role of *ABI1* gene dosage and expression in the lung metastasis process which may model metastatic potential of CNA and gene expression of *ABI1* in patient’s primary breast tumors (Fig. 1A), consistent with histologic high‐aggressive breast cancers (Fig. 1B), and basal‐like subtype (Fig. 1D)—hallmarks of high aggressive invasive breast cancer with polyclonal metastases potential. Also, our experimental findings consist of high ABI1 protein expression in human invasive breast carcinoma associated with high risks of tumor recurrence and overall survival (Fig. 2, Figs. S1 and S3, [32]).

It was observed that protein interaction combinations of WASF3 with some members of WAVE complex and RAC1 are responsible for breast cancer aggressiveness and metastasis [22]. In our study, we found an association of WASF3 and some other WAVE complex components (that are part of the prognostic signature) with invasive breast cancer that molecular pattern is associated with aggressive (basal‐like) breast cancer subtype. Interestingly, heterogeneity and instability of Wave complexes without Abi1 protein could contribute to the heterogeneity in latency, the size and number of lung metastatic lesions as observed in *Wasf3* KO mice [22].

Our data adhere to previously published findings regarding the impact of ABI1 protein in driving aggressive mammary oncogenesis in mouse xenograft models of breast cancer [17, 34]. ABI1 has been cited in several cancer types, such as ovarian cancer [29, 64], hepatocellular carcinoma [65], and colorectal carcinoma [66]. Notably, all studies to date examined the role of ABI1 in breast cancer using cancer cell lines. This is the first genetic study examining the role of *Abi1 in vivo* using the mouse model of aggressive breast cancer. The critical role of *Abi1* in the lung metastasis in the mouse not only provides preclinical evidence for the role of *Abi1* in metastatic progression but also supports *ABI1*‐based 7‐gene prognostic signature as both a prognostic marker and a prospective therapeutic target.

Univariate and multivariate analyses and comparison of Kaplan–Meier survival curves generated with our *ABI1* gene expression signature to those generated with either estrogen receptor (ESR) or lymph node status reveal that our gene signature is indeed a more robust prognostic predictor than other clinically used variables and could lead to better treatment selection.

## 5 Conclusion

Our findings indicate the significant predictive value of the *ABI1*‐based 7‐gene prognostic signature derived from primary tumors in the metastatic risk of breast cancer patients. Moreover, targeting *ABI1* may provide a beneficial therapeutic effect in preventing metastases.

## Conflict of interest

The authors declare no conflict of interest.

## Author contributions

LK designed and interpreted the experimental results of the study. VAK designed bioinformatic and statistical analyses and interpreted the results of this study; VAK and LK wrote the final version of the manuscript. AG, CP, and VAK performed the analyses. AR performed all animal experiments and cowrote the paper with LK and VAK. BAP performed qPCR analysis. TC completed the pathological assessment of tumors. IB analyzed TCGA breast tumor mutation data sets. MK helped with experimental design in mice. VAK, GB, and AS contributed to the interpretation and discussion of the clinical significance of the data presented in this paper.

## Consent for publication

All authors have agreed to publish this manuscript.

### Peer Review

The peer review history for this article is available at https://publons.com/publon/10.1002/1878‐0261.13175.

## Supporting information


**Fig S1**. Annotation discrepancies and cluster analysis.
**Fig S2**. Example of KS‐weighted means batch effect correction and its effect on survival analysis.
**Fig S3**. Risk‐predicting ability of individual members of the ABI1‐WAVE signature in disease‐free survival (DFS).
**Fig S4**. Risk‐predicting ability of individual members of the ABI1‐WAVE signature in distant metastasis‐free survival (DMFS).
**Fig S5**. Commonly used clinical variables are insufficient for robust patient risk stratification.
**Fig S6.** Survival predictive analysis (RFS time) at transcription and protein level suggests a pro‐oncogenic role of ABI1 in BC progression and outcome.
**Fig S7.** The implementation of 2D‐DDg survival prediction to Rosetta data (DFS and DMFS).

**Table S1**. Gene list for prognostic signatures and associated probes/probsets represented by Rosetta (Merk) and Affymetrix U133‐A & B microarrays.
**Table S2**. Results of 1D DDg survival prediction (DFS and DMFS) for Rosetta and Metadata sets.
**Table S3.** 1D‐DDg by ABI1 expression level suggests significant metastasis events enrichment in a high‐risk group (DMFS time).
**Table S4.** Survival significant prognostic genes encoding WAVE complex and *NDEL* gene are correlated with expression of ABI1. METABRIC breast cancer Dataset (*n* = 1904).
**Table S5.** Significance of association between the survival stratification grouping (by DMFS) and metastatic events (A, B) and our estimations of the probability of metastasis risk events (C,D).
**Table S6.** Abi1‐based signature predicts distant metastatic events more accurately than commonly used clinical factors. Cox univariate and multivariate hazards proportional models analysis compares the ABI1‐based 7‐gene metastasis risk classifier (low, moderate, high risks by SVWg), ESR status (ER(+), ER(‐)), and lymph node status (LN(+), LN(‐)) to predict metastatic events in the Rosetta cohort.
**Table S7.** Basal‐like vs. luminal cell type markers in primary breast tumors of Abi1 KO mice.
**Table S8.** Mouse breast pathology Ki67 and grading data.
**Table S9.** Data and statistical analysis of pulmonary node metastases size characteristics.
**Table S10.** Primary tumor size kinetic data.

## Data Availability

All data generated or analyzed during this study are included in this manuscript.
