# Supplementary material for: ABI1‐based expression signature predicts breast cancer metastasis and survival
Source: Mol Oncol. 2022 Jan 26;16(14):2632–57. doi: 10.1002/1878-0261.13175 (PMC9297774; doi:10.1002/1878-0261.13175)
Supplement: Supplementary file 1 — Fig S1. Annotation discrepancies and cluster analysis. Fig S2. Example of KS‐weighted means batch effect correction and its effect on survival analysis. Fig S3. Risk‐predicting ability of individual members of the ABI1‐WAVE signature in disease‐free survival (DFS). Fig S4. Risk‐predicting ability of individual members of the ABI1‐WAVE signature in distant metastasis‐free survival (DMFS). Fig S5. Commonly used clinical variables are insufficient for robust patient risk stratification. Fig S6. Survival predictive analysis (RFS time) at transcription and protein level suggests a pro‐oncogenic role of ABI1 in BC progression and outcome. Fig S7. The implementation of 2D‐DDg survival prediction to Rosetta data (DFS and DMFS). [file MOL2-16-2632-s002.pdf]

Supplementary Figures

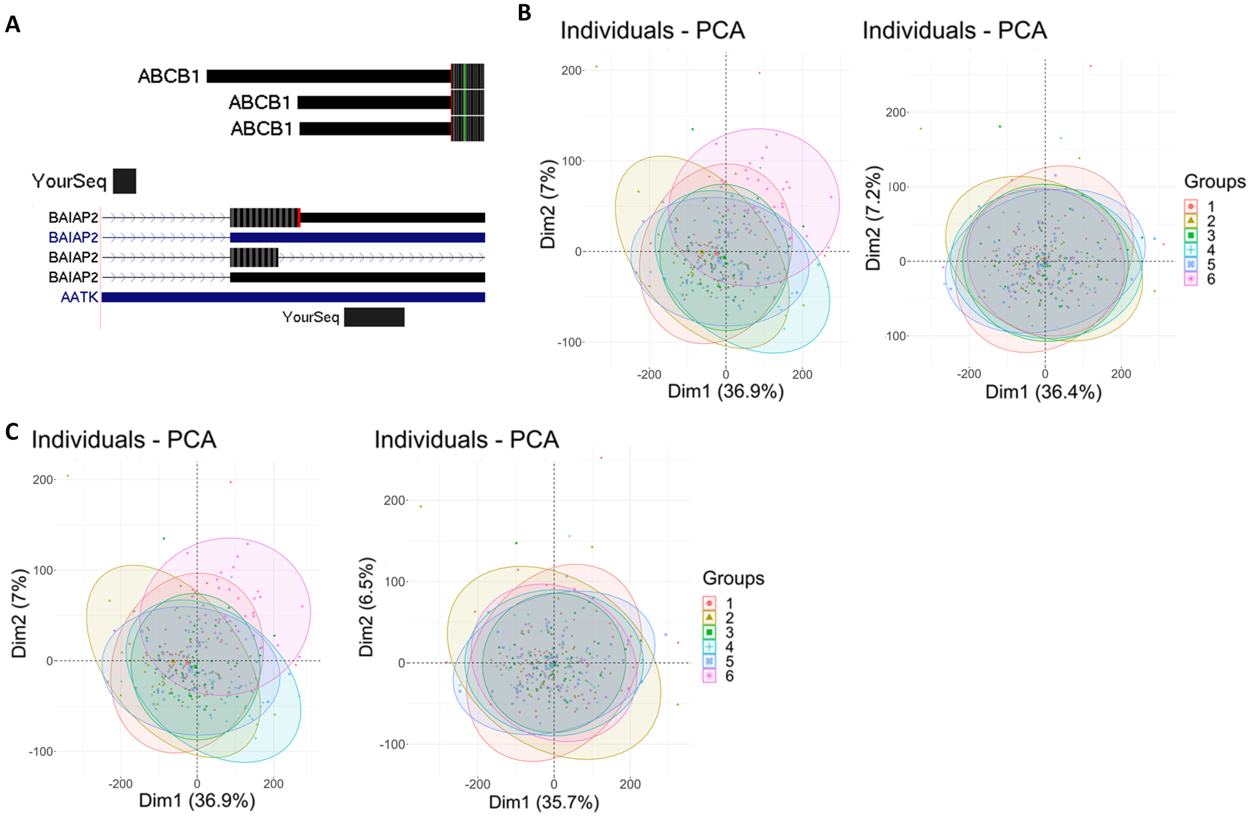

Figure S1. Annotation discrepancies and cluster analysis. Examples of false-positive and multiple mapping, top and bottom respectively (**A**). Here, instances are shown of incorrection annotation in the Rosetta probe set. PCA analysis of the microarray expression dataset pre-batch effect correction and pos-batch effect correction, left and right respectively, utilizing ComBat (**B**) and KS-weighted mean (**C**) Batch correction methods. Here, we show that before batch effect correction, variation exists between groups. Utilizing ComBat and KS-weighted mean correction provides similar results.

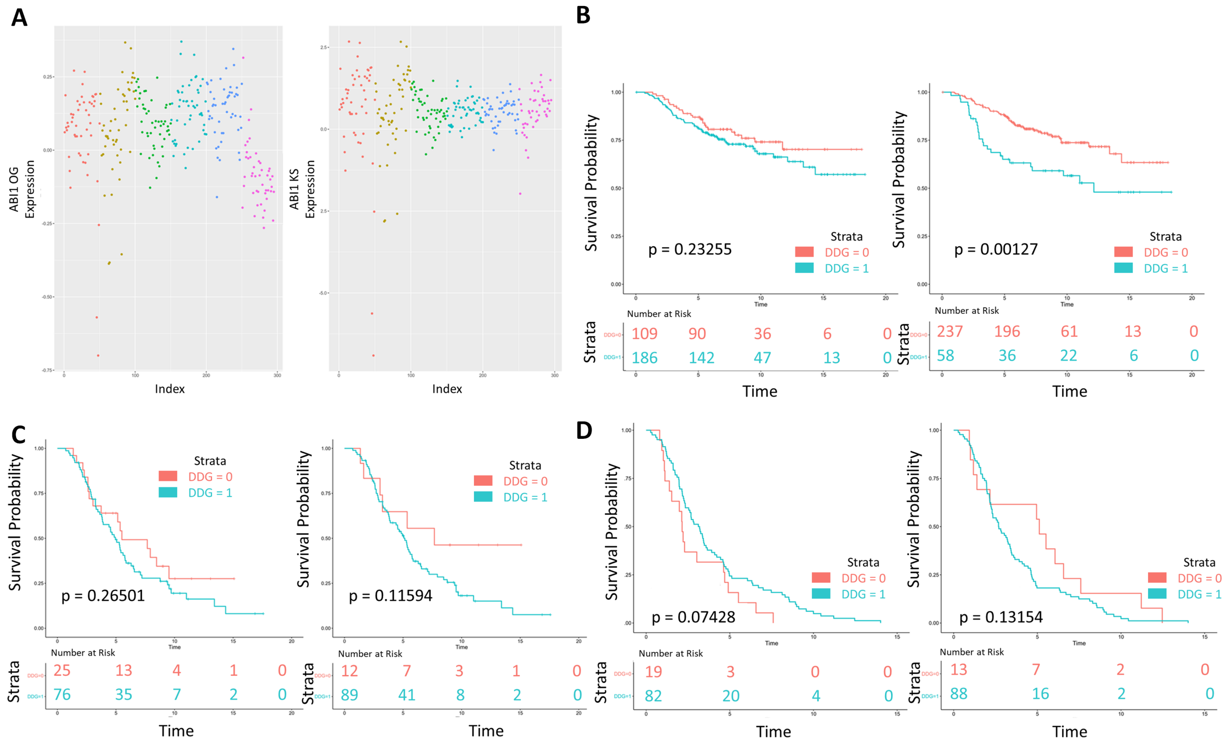

Figure S2. Example of KS-weighted means batch effect correction and its effect on survival analysis. Distributions of ABI1 expression values for each group pre-batch effect correction and post-batch effect correction, left and right respectively **(A)**. Here, we show increased consistency of ABI1 expression values between groups. Survival analysis of survival time and event death of original dataset and KS-corrected dataset including all patients left and right respectively **(B)**. Survival analysis of survival time and event death of original dataset and KS-corrected dataset in only patients with metastasis left and right respectively **(C).** Survival analysis of metastasis time and metastasis event of the original dataset and KS-corrected dataset in only patients with metastasis, left and right respectively **(D).** In the case of (**B**) and (**C**), KS-weighted means batch effect correction results in better significance. For D, KS-weighted means batch effect correction provides results that adhered to findings in the literature while the original dataset did not.

A

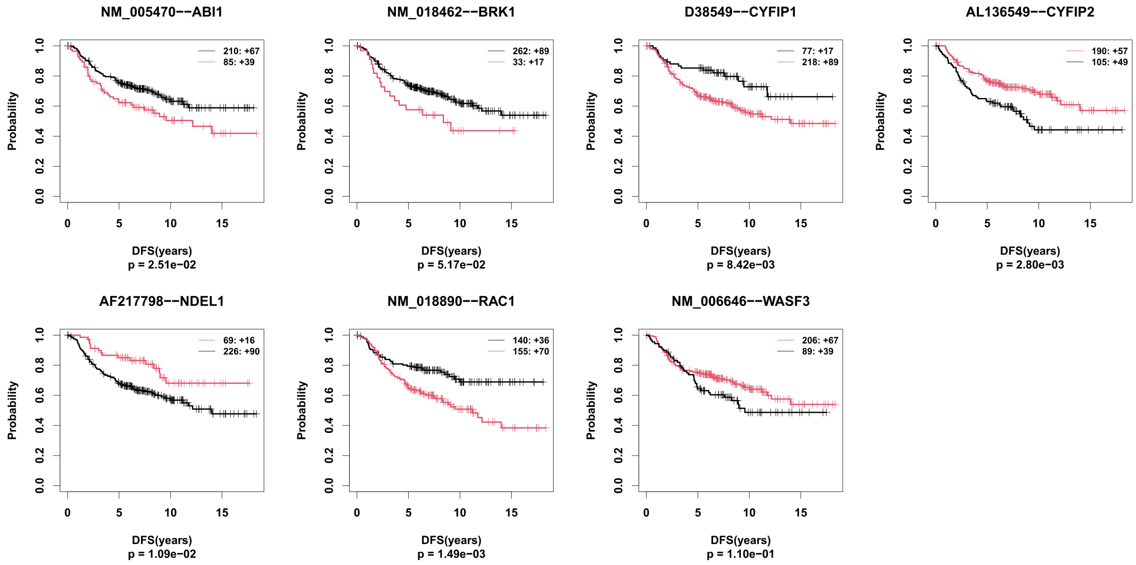

B

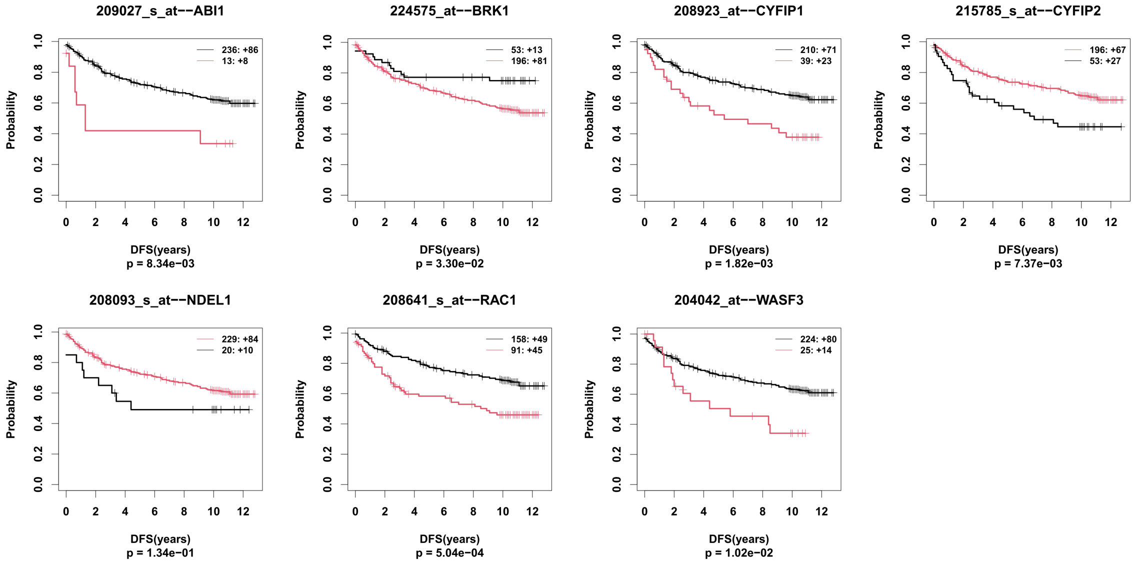

Figure S3. Risk-predicting ability of individual members of the ABI1-WAVE signature in disease-free survival (DFS). Kaplan-Meier survival curves depict the survival associated with patients stratified into low-risk and high-risk groups according to the ABI1-WAVE signature in the Rosetta (**A**) and MetaData (**B**) datasets. In all plots, black color is associated with low-risk and red color is associated with high-risk.

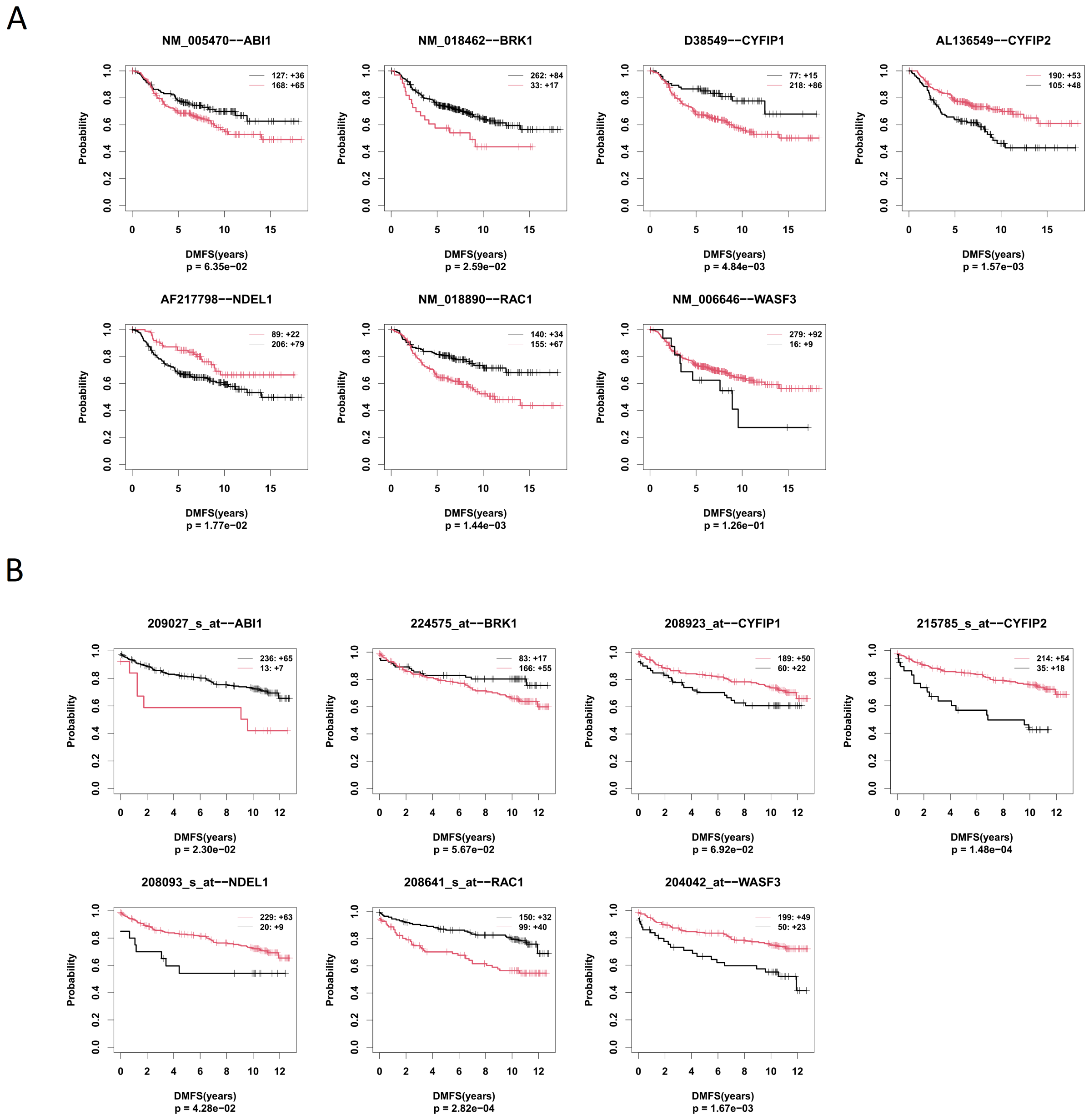

Figure S4. Risk-predicting ability of individual members of the ABI1-WAVE signature in distant-metastasis free survival (DMFS). Kaplan-Meier survival curves depict the survival associated with patients stratified into low-risk and high-risk groups according to the ABI1-WAVE signature in the Rosetta (**A**) and MetaData (**B**) datasets. In all plots, black color is associated with low-risk and red color is associated with high-risk.

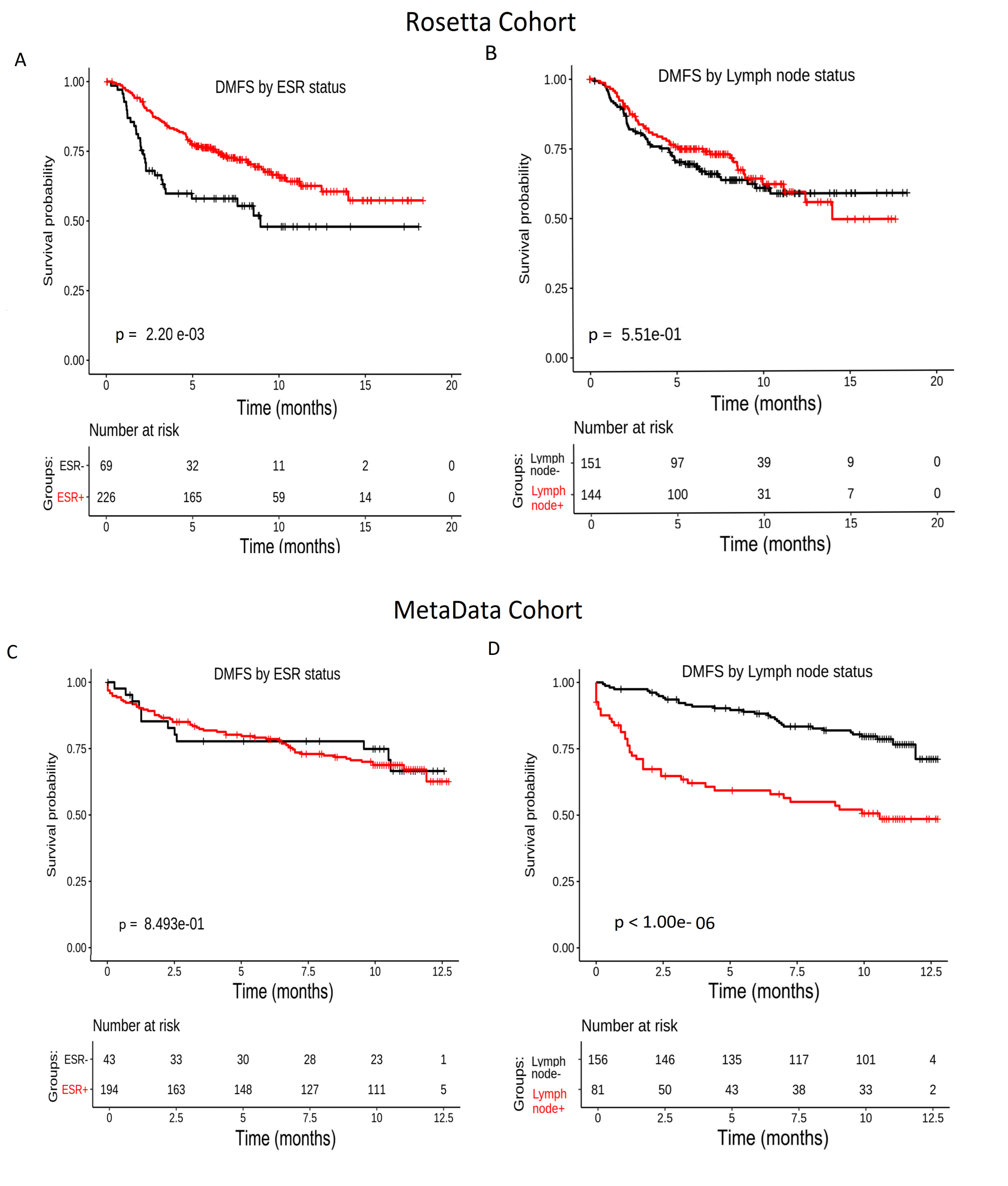

Figure S5. Commonly used clinical variables are insufficient for robust patient risk stratification. Kaplan-Meier survival curves for the Rosetta dataset were stratified based on (a) estrogen receptor (ESR) status (red: positive vs. black: negative; Log-rank p=0.0022) and (b) lymph node status (red: positive vs. black: negative; Log-rank p=0.52). Kaplan-Meier survival curves for the Rosetta dataset were stratified based on

(c) estrogen receptor (ESR) status (red: positive vs. black: negative; Log-rank p=0.0.85) and (d) lymph node status (red: positive vs. black: negative; Log-rank p < 0.001).

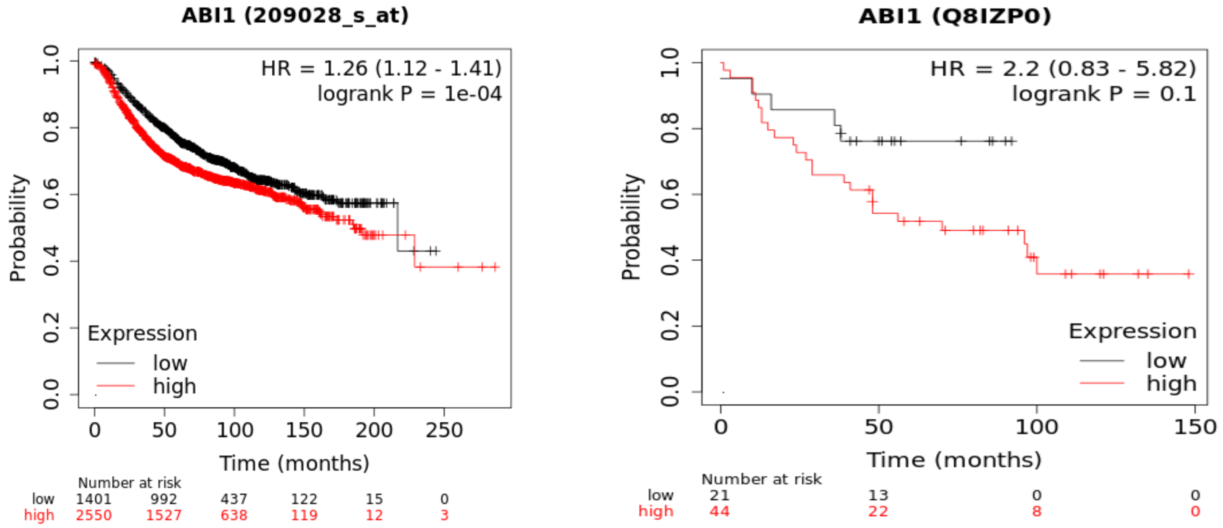

Figure S6. Survival predictive analysis (RFS time) at transcription and protein level suggests a pro-oncogenic role of ABI1 in BC progression and outcome. (**A**) Microarray mRNA expression dataset of the 3951 BC patients suggests a pro-oncogenic role of ABI1 in BC progression and outcome. p=0.0001, Log rank test; FDR=5%, Median survival time (months): low expression: 216.7; high expression: 185.2. Expression cut-off value: 746, expression range: 121 - 4621. Dataset source and method:https://kmplot.com/analysis/index.php?p=service&cancer=breast. (**B**) Protein level analysis support ABI1 as a survival prognostic marker in BC patient samples. Survival prediction analysis (Data: GEO/NCBI GSE39004) (2): http://kmplot.com/analysis/index.php?p=service&cancer=breast_protein

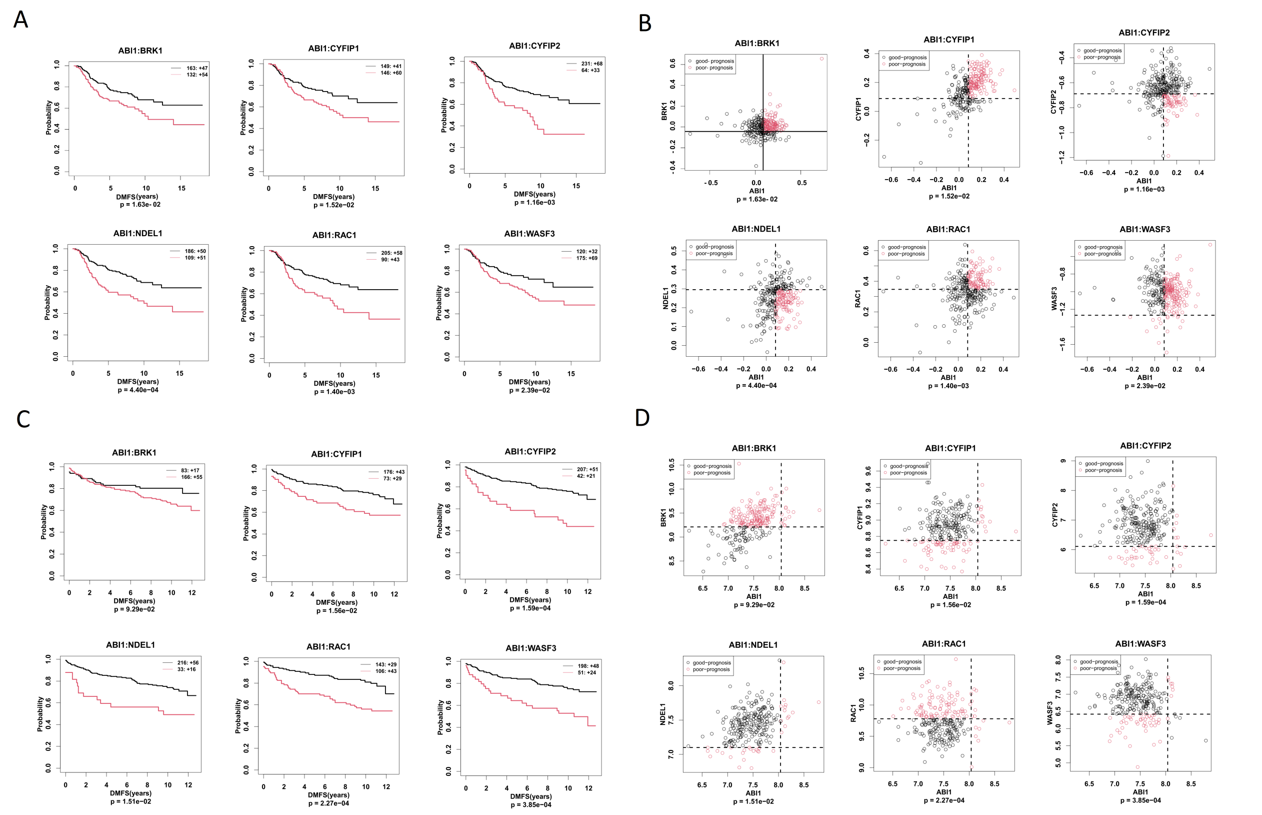

Figure S7. The implementation of 2D-DDg survival prediction to Rosetta data (DFS and DMFS). Panels (**A**) and (**C**) show the K-M function plots for low- and high-risk groups defined by *ABI1* expression paired with the expression of our other 6 genes (as potential interaction partners) for DFS and DMFS respectively. Panels (**B**) and (**D**) provide a visual presentation of bi-bivariate distributions of the expression data in the case of DFS and DMFS respectively. The scatter plots give a visual representation of the separation of patients according to the individual cut-off values associated with each gene in the synergistic gene pairs, for Rosetta (**B**) and MetaData (**D**) datasets. Each circle represents a tumor sample; circles with red outlines are associated with relatively high risk and circles with black outlines are associated with lower-risk survival outcome groups. In (**A**) and (**C**) the red color indicates the survival time of the high-risk group, while the color black indicated the survival function of the low-risk group. In panels (**B**) and (**D**), the points colored with red correspond to patients in the high-risk group, and the black points refer to patients in the low-risk group.

References:

1. Pereira B, Chin SF, Rueda OM, Vollan HK, Provenzano E, Bardwell HA*, et al.* The somatic mutation profiles of 2,433 breast cancers refines their genomic and transcriptomic landscapes. *Nat Commun* 2016;**7**:11479 doi 10.1038/ncomms11479.

2. Tang W, Zhou M, Dorsey TH, Prieto DA, Wang XW, Ruppin E*, et al.* Integrated proteo-transcriptomics of breast cancer reveals globally increased protein-mRNA concordance associated with subtypes and survival. *Genome Med* 2018;**10**(1):94 doi 10.1186/s13073-018-0602-x.
